# Advanced solvent signal suppression for the acquisition of 1D and 2D NMR spectra of Scotch Whisky

**DOI:** 10.1002/mrc.4621

**Published:** 2017-06-29

**Authors:** Will Kew, Nicholle G.A. Bell, Ian Goodall, Dušan Uhrín

**Affiliations:** ^1^ EastCHEM School of Chemistry University of Edinburgh King's Buildings, David Brewster Road Edinburgh EH9 3FJ UK; ^2^ The Scotch Whisky Research Institute The Robertson Trust Building, Research Avenue North, Riccarton Edinburgh EH14 4AP UK

**Keywords:** ^1^H, ^13^C, alcoholic beverages, complex mixture, NMR, Scotch Whisky, solvent suppression

## Abstract

A simple and robust solvent suppression technique that enables acquisition of high‐quality 1D ^1^H nuclear magnetic resonance (NMR) spectra of alcoholic beverages on cryoprobe instruments was developed and applied to acquire NMR spectra of Scotch Whisky. The method uses 3 channels to suppress signals of water and ethanol, including those of ^13^C satellites of ethanol. It is executed in automation allowing high throughput investigations of alcoholic beverages. On the basis of the well‐established 1D nuclear Overhauser spectroscopy (NOESY) solvent suppression technique, this method suppresses the solvent at the beginning of the pulse sequence, producing pure phase signals minimally affected by the relaxation. The developed solvent suppression procedure was integrated into several homocorrelated and heterocorrelated 2D NMR experiments, including 2D correlation spectroscopy (COSY), 2D total correlation spectroscopy (TOCSY), 2D band‐selective TOCSY, 2D *J*‐resolved spectroscopy, 2D ^1^H, ^13^C heteronuclear single‐quantum correlation spectroscopy (HSQC), 2D ^1^H, ^13^C HSQC‐TOCSY, and 2D ^1^H, ^13^C heteronuclear multiple‐bond correlation spectroscopy (HMBC). A 1D chemical‐shift‐selective TOCSY experiments was also modified. The wealth of information obtained by these experiments will assist in NMR structure elucidation of Scotch Whisky congeners and generally the composition of alcoholic beverages at the molecular level.

## 1 INTRODUCTION

Scotch Whisky is a culturally significant and high‐value commodity. Chemical analysis of Scotch Whisky is therefore essential to gain a better understanding of the production processes and maturation chemistry^1^, ^2^ as well as for addressing food safety challenges,^3^, ^4^, ^5^ and authenticity concerns.^6^, ^7^, ^8^, ^9^, ^10^, ^11^ Scotch Whisky is produced by the fermentation of a cereal source, distillation to below 94.8% (*v*/v) ethanol, and maturation in oak casks in Scotland for a minimum of 3 years.^12^ Its production follows strict guidelines outlined in the Scotch Whisky Regulations (2009),^13^ and yet every distillery produces a unique spirit.

Traditionally, chemical analysis of whisky has used standard mass spectrometry (MS) techniques, in particular gas chromatography (GC)‐ and liquid chromatography (LC)‐MS, to identify and quantify its compounds, referred to as congeners.^12^, ^14^ These methods are labour intensive to establish, often compound specific, and require several instruments and skilled operators to prepare the samples and interpret the results.

Lately, several studies have been carried out using high‐end MS techniques to characterise whisky.^8^, ^15^ Our recent Fourier transform ion cyclotron resonance (FT‐ICR) MS study^15^ revealed the presence of thousands of compounds in a large set of curated whisky samples. Even though we have managed to associate over 72% of MS peaks with unique molecular formulae, the structures of these compounds and their concentrations cannot be determined by this technique, which in addition fails to detect many low molecular weight congeners prevalent in whisky samples.

NMR spectroscopy, on the other hand, represents a means to detect, identify, and quantify all major components of whisky by a single technique. It is a nonselective and nondestructive method, allowing for simultaneous profiling of different compound classes, albeit with a limited dynamic range and sensitivity. Accepting the NMR detection limits, whisky is still a complex mixture that, in addition to this complication, presents additional challenges. In common with other alcoholic beverages, whisky primarily comprises of two protonated compounds—water and ethanol. Their signals dominate the standard ^1^H spectra of spirits and need to be suppressed to allow observation of important, but lower abundance, congeners.

Numerous pulsed field gradient (PFG)‐based methods have been developed for solvent suppression, most notably H_2_O in biological samples.^16^, ^17^, ^18^, ^19^ Signal suppression of H_2_O is relatively straightforward, as its NMR signal is a singlet. Solvent suppression is more challenging when several multiplets need to be suppressed—a case typical for LC‐NMR using nondeuterated solvents.^20^, ^21^ In these situations, *n‐*solvent peaks are peak picked in a 1D ^1^H spectrum, and appropriate‐shaped pulses are generated for their suppression. These techniques, when performed in automation, are limited by a few factors, mostly due to the nonoptimised suppression methodology. Significantly, they will not decouple the ^13^C satellites of a carbon‐bonded solvent's proton by default. Similarly, the presence of ethanol makes the solvent suppression in whisky samples challenging. The ^1^H NMR spectrum of ethanol consists of a triplet and quartet, alongside a residual –OH signal, which under certain circumstances appears separately from the water signal. In addition, the nonlabile ethanol protons show ^13^C satellite signals, which are not insignificant relative to the concentration of the congeners. A solution containing 40% ethanol has ethanol concentration of 6.85 M. The ^13^C^12^CH_5_OH isotopomers of ethanol with natural abundance of ^13^C are therefore present at approximately 75 mM. For comparison, a major whisky congener, isobutanol, occurs at a concentration of approximately 4 mM in malt Scotch Whisky.^14^ It is clear that any successful attempt to suppress ethanol must also remove its carbon satellites during the solvent suppression.^21^

In this work, we present an automated ethanol/water solvent suppression methodology allowing for the acquisition of high resolution, high sensitivity 1D ^1^H NMR spectra of whisky with minimal sample preparation. The developed 1D method uses the well‐established 1D NOESY pulse sequence, which in combination with signal presaturation yields very good water suppression,^22^ as documented by its widespread use in NMR studies of biofluids.^23^, ^24^, ^25^, ^26^ This solvent suppression method is also used as the basis for even more challenging solvent suppression in 2D homonuclear and heteronuclear NMR experiments. Several examples of these experiments are presented here.

## 2 MATERIALS AND METHODS

Samples were analysed in 5 mm Wilmad 535‐PP‐7 Precision NMR tubes rated for 600 MHz. D_2_O (99%), acetic acid‐d_4_ (99.5%), and sodium acetate‐d_3_ (99%) were acquired from Sigma‐Aldrich Co. Samples (500 μL) of Scotch Whisky, provided by the Scotch Whisky Research Institute, were mixed with D_2_O/sodium acetate/acetic acid buffer solution (100 μL) containing DSS‐d_6_ as an internal standard. The final concentrations of buffer and internal standard were 25 and 1 mM, respectively. Samples of very high ethanol strength (>80% *v*/v) were diluted using ultra high quality (UHQ) water (1:1) before mixing with the buffer solution. All spectra were acquired at 300.0 K.

NMR experiments were developed and implemented on 4‐channel Avance III HD 600 and 800 MHz Bruker spectrometers equipped with a TCI cryoprobe with automated matching and tuning and a 16‐slot sample changer (600 MHz only). All spectra presented here were acquired at 600 MHz unless otherwise noted. Acquisition of a 1D ^1^H spectrum is preceded by the acquisition of three preliminary spectra in automation using IcoNMR and Python scripting. The 1D ^1^H spectra were acquired in full automation, whereas subsequent 2D experiments were acquired in manual mode.

The first preliminary experiment serves to locate the exact resonance frequency of water and to calibrate 90° ^1^H pulses on the first and third channels. The water signal is located using a 360° pulse in a single scan experiment. The backwards linear prediction of the first 4,096 points used during the processing removes the broad component of the water signal, allowing for the accurate determination of the frequency of the sharp component. The determined value is then used to irradiate the water signal on the 3rd channel. Only the first 512 points are back predicted for samples with a high ethanol concentration, due to fast decay of their water signal. An example of the spectrum acquired from this experiment is provided in Figure S1.

The second preliminary experiment determines the exact chemical shift of CH_2_ and CH_3_ signals of ^12^C isotopomers of ethanol by establishing the corresponding chemical shift in ^13^C isotopomers in a nonrefocused reverse INEPT^27^, ^28^ experiment. This approach was chosen as the radiation damping prevents accurate determination of the chemical shifts of ethanol protons in ^12^C isotopomers. The reverse insensitive nuclei enhanced by polarization transfer (INEPT) spectrum is acquired using acquisition and relaxation times of 6.06 and 4 s, four dummy scans and 16 scans with ^1^H and ^13^C carrier frequencies of 2.2 and 40.0 ppm, respectively. The free induction decay (FID) is zero‐filled once and Fourier transformed using exponential broadening of 0.2 Hz. The antiphase satellite ethanol signals are peak picked, and the chemical shifts of CH_2_ and CH_3_ signals of ^12^C isotopomers are calculated by adding 1.53 and 1.11 Hz to the corresponding chemical shifts of ^13^C isotopomers, respectively, to compensate for the ^13^C isotope shift. A frequency‐modulated rectangular‐shaped pulse, *EthanolShape*, is generated starting from a square pulse (1000 points). This pulse inverts the on‐resonance CH_2_ protons and the off‐resonance CH_3_ protons. Its exact length, to be around 50 ms, is calculated to allow 2π*n* rotation for the off‐resonance CH_3_ protons. This experiment also stores the centre position of the CH_2_ signal as the carrier frequency for the final experiment. The power used for this pulse corresponds to γB_1_/2π equal to 28 Hz. An example of the spectrum acquired from this experiment is provided in Figure S2.

The third preliminary experiment produces a single scan 1D ^13^C spectrum acquired with inverse‐gated ^1^H decoupling. The spectrum is acquired using an acquisition time of 2.04 s with ^1^H and ^13^C carrier frequencies set to 2.20 and 40 ppm, respectively. The spectrum is peak picked and the positions of the ethanol carbon signals are used to calculate a shaped pulse, *CarbonShape*, for decoupling of the ethanol satellites in the final experiment. The ^13^C carrier is placed exactly in the middle of the CH_2_ and CH_3_^13^C chemical shifts. A 180° rectangular cosine modulated shaped pulse consisting of around 500 points and length approximately 2.5 ms is generated—the exact number of points and length is optimised per sample. Its exact length is calculated to allow 2π*n* rotation for both resonances. The power level was set to γB_1_/2π equal to 333 Hz, and the pulses were phase cycled using m4p5 scheme, where m4 and p5 are {0°, 0°, 180°, 180°} and {0°, 150°, 60°, 150°, 0°}, respectively.^29^, ^30^ An example of the spectrum acquired from this experiment is provided in Figure S3.

The final experiment imports the various offsets, calculated pulse shapes, and power levels to acquire a 1D ^1^H spectrum with water and ethanol suppression. For water presaturation, the power level was set to γB_1_/2π equal to 20 Hz. The receiver gain is set to 45.2, and spectra are obtained using four dummy scans and 32 scans. The FID is acquired with digitised sampling of 128 k time domain points over 16 ppm, yielding the acquisition time of 6.82 s; a 4.5‐s relaxation/presaturation delay was used. The FID was zero filled once and Fourier transformed using an exponential line broadening of 0.2 Hz. An example of the spectrum acquired from this experiment is provided in Figure S4.

These experiments were performed in automation and take approximately 15 min per sample, including loading and shimming. In‐house Python scripts were used for batch processing of spectra including phasing and baseline corrections.

The pulse sequence of a magnitude double quantum filter (DQF) COSY experiment (Bruker pulse sequence *cosygpppqf*) was modified to include solvent signal presaturation during a 3‐s relaxation delay as outlined for the acquisition of 1D ^1^H NMR spectra. The 2D COSY spectra were acquired using 1,024 and 4,096 complex points in *F*_1_ and *F*_2_ using spectral widths of 10 and 13 ppm in *F*_1_ and *F*_2_ yielding *t*_1_ and *t*_2_ acquisition times of 85.2 and 262 ms, respectively. Four scans were accumulated per one increment resulting in the overall acquisition time of 3 hr 52 min. A forward linear prediction to 2,048 points was used in *F*_1_ and zero filling to 8,192 was applied in *F*_2_. A sine square window function was used for apodization prior to Fourier transformation in both dimensions.

Similar modifications to those described for the COSY experiments were implemented into a phase‐sensitive TOCSY experiment using a z‐axis decoupling in the presence of scalar interactions (DIPSI)‐2 spin‐lock^31^ starting from the Bruker pulse sequence *dipsi2esgpph*. A 40‐ms spin‐lock was applied at γB_1_/2π of 10 kHz. The PFGs surrounding the DIPSI spin‐lock were applied at 1% and 3%. The spectrum was acquired using 768 and 4,096 complex points in *F*_1_ and *F*_2_ using spectral widths of 10 and 13 ppm in *F*_1_ and *F*_2_ yielding *t*_1_ and *t*_2_ acquisition times of 63.9 and 262 ms, respectively. Sixteen scans were accumulated per one increment resulting in the overall acquisition time of 8 hr 8 min. A forward linear prediction to 2,048 points was used in *F*_1_ and zero filling to 8,192 was applied in *F*_2_. A cosine square window function was used for apodization prior to Fourier transformation in both dimensions.

A band‐selective 2D TOCSY experiment^32^ was designed starting from the Bruker pulse sequence *dipsi2gpphzs*. Two 2D band‐selective TOCSY spectra were acquired focusing on methyl and methylene regions centred at 0.741 and 1.289 ppm, respectively, using the relaxation time of 2 s, mixing time of 80 ms, and eight scans per increment. As these regions do not include solvent signals, no signal suppression was necessary. A zero‐quantum suppression was achieved using 20 ms CHIRP pulse with a simultaneous PFG (11%) before and after mixing according to Thrippleton et al.^33^

The methyl focussed experiment was acquired using the following parameters: 128 and 4,096 complex points in *F*_1_ and *F*_2_, respectively, spectral widths of 0.083 and 7.0 ppm in *F*_1_ and *F*_2_, yielding *t*_1_ and *t*_2_ acquisition times of 487 ms and 1.28 s, respectively. The overall acquisition time was 1 hr and 16 min. A forward linear prediction to 256 points was used in *F*_1_ followed by zero filling to 512 points. Zero filling to 8,192 was applied in *F*_2_. A cosine square window function was used for apodization prior to Fourier transformation in both dimensions.

The methylene focussed band‐selective TOCSY experiment was acquired using the following parameters: 576 and 4,096 complex points in *F*_1_ and *F*_2_, respectively, spectral widths of 0.832 and 8.4 ppm in *F*_1_ and *F*_2_, yielding *t*_1_ and *t*_2_ acquisition times of 576 and 405 ms, respectively. The overall acquisition time was 4 hr and 14 min. A forward linear prediction to 1,024 points was used in *F*_1_. A zero filling to 8,192 was applied in *F*_2_. A cosine square window function was used for apodization prior to Fourier transformation in both dimensions.

A magnitude mode *J*‐resolved experiment (Bruker pulse sequence *jresqf*) was modified to include the presaturation elements as above. Spectra were acquired using 96 and 4,096 complex points in *F*_1_ and *F*_2_, respectively. Sixteen scans were acquired per increment using spectral widths of 40 Hz and 13 ppm in *F*_1_ and *F*_2_, yielding *t*_1_ and *t*_2_ acquisition times of 0.26 and 1.2 s, respectively. The overall experimental time was 2 hr and 20 min. A forward linear prediction to 256 points was used in *F*_1_, followed by zero filling to 512 points. A zero filling to 8,192 was applied in *F*_2_. A sine square window function was used for apodization prior to Fourier transformation in both dimensions.

A 1D chemical‐shift‐selective filter (CSSF) TOCSY experiment^34^ using DIPSI‐2 spin‐lock^31^ and zero‐quantum suppression^33^ was modified to include solvent signal presaturation during the relaxation delay. The spin‐lock at γB_1_/2π of 10 kHz was applied from 10 to 160 ms. Selective inversion of a selected proton was achieved using a 40‐ms Gaussian pulse applied on Channel 1 after switching to the desired frequency from the frequency of the CH_2_ ethanol signal used during the presaturation. The spectra were obtained with 32 increments (2.6 ms) of the CSSF (total length 83.2 ms) with two scans each. The 1D TOCSY spectra were acquired using 32 k time domain points and a spectral width of 14 ppm, yielding an acquisition time of 1.46 s; a 2.0‐s relaxation delay was used. The FID was zero filled once and Fourier transformed using exponential line broadening of 0.3 Hz.

An echo/anti‐echo DEPT‐edited ^1^H, ^13^C HSQC experiment, Bruker pulse sequence *hsqcedetgpsisp2.3* was modified to include explicit adiabatic bilevel decoupling^35^, ^36^, ^37^, ^38^, ^39^, ^40^, ^41^ and the solvent suppression as described for the acquisition of 1D ^1^H NMR spectra. Standard Bruker broad‐band CHIRP ^13^C pulses were used including 500 and 2,000 μs long inversion and refocusing pulses, respectively. Analogous 2D ^1^H, ^13^C HSQC‐TOCSY with DIPSI‐2 spin lock but without the multiplicity selection was also modified (standard Bruker pulse program *hsqcdietgpsisp.2*) to include signal suppression. The heterocorrelated spectra were acquired using 1,024 and 3,072 complex points in *F*_1_ and *F*_2_, respectively. Eight scans (HSQC) or 16 scans (HSQC‐TOCSY) were acquired per increment using spectral widths of 15 and 165 ppm in ^1^H and ^13^C dimensions yielding *t*_1_ and *t*_2_ acquisition times of 21 and 176 ms, respectively. The ^13^C carrier frequency was set to 80 ppm. The spectrum was zero filled to 8,192 and 2,048 points, and the cosine square window function was used before Fourier transformation; a 40‐ms spin‐lock was applied at γB_1_/2π of 10 kHz in the HSQC‐TOCSY experiment.

A constant‐time phase‐sensitive gradient‐selected ^1^H, ^13^C HMBC experiment with twofold low‐pass *J*‐filter to suppress one‐bond correlations and no decoupling during acquisition,^42^, ^43^ Bruker standard pulse program *hmbcctetgpl2nd*, was modified to include signal presaturation during the relaxation delay, as described above. Additional ethanol CH_2_ and water signal saturation (γB_1_/2π = 28 Hz) were applied during the long‐range evolution delay optimised for ^n^ *J*_CH_ of 6 Hz. A two‐step low pass *J*‐filter was applied optimised for the ^1^J_CH_ couplings of ethanol (125.7 and 142.6 Hz). The 90° ^13^C pulses of the filter were implemented as composite 90°_x_‐90°_y_ pulses. The 2D ^1^H, ^13^C HMBC spectra were acquired using 910 and 4,096 complex points in *F*_1_ and *F*_2_, respectively. Sixteen scans were acquired using spectral widths of 14 and 200 ppm in ^1^H and ^13^C dimensions yielding *t*_1_ and *t*_2_ acquisition times of 15 and 243 ms, respectively. The ^13^C carrier frequency was set to 100 ppm. The spectrum was zero filled to 8,192 and 4,096 points, and the cosine square window function was used before Fourier transformation. Full example spectra for all experiments are included in Data S1.

## 3 RESULTS AND DISCUSSION

### 3.1 Sample preparation

A sample preparation protocol was designed to minimise time and costs to allow high‐throughput acquisition of spectra. Scotch Whisky is an acidic solution (pH ca. 4), with acidity increasing during maturation.^14^ To minimise the chemical shift changes due to pH variations between samples, a buffer solution was added to each sample. Deuterated sodium acetate/acetic acid buffer (25 mM) was used as acetic acid is naturally occurring in whisky at a typical concentration of 1.5 mM, and its pK_a_ is close to whisky's natural pH. The residual signal of the undeuterated acetic acid (0.5%, 125 μM) at 2.08 ppm thus contributes to the naturally occurring signal. The final buffer concentration of 25 mM was decided upon as a compromise for achieving sufficient buffering capacity, whilst minimising the reduction in sensitivity of cryoprobes caused by salt.^44^ Using these conditions, the pH variations were limited to few hundredths of pH units. In addition, it was observed that changes in ethanol strength also contributed to the movement of signals, but no attempt was made to equalise it, as this would substantially increase the complexity of sample preparation.

### 3.2 Ethanol and water suppression in whisky samples

**Figure 1:**
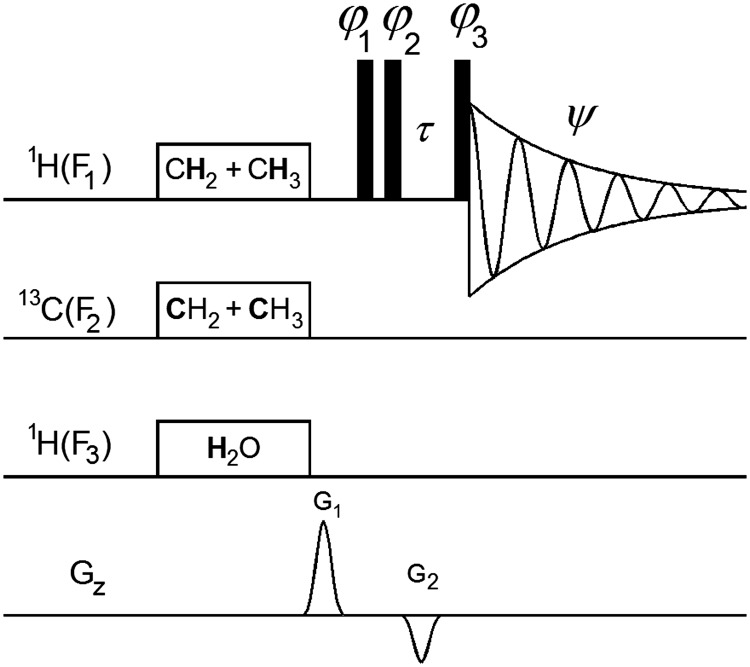
Pulse sequence for simultaneous suppression of water, ethanol, and ^13^C ethanol satellites in whisky samples. Narrow‐filled rectangles represent 90° pulses. The 1‐ms CHIRP‐shaped pulsed field gradients (PFGs) were applied at G_1_ = 50% and G_2_ = −11% followed by a 200‐μs gradient recovery delay, resulting in a τ period of 1.2 ms. The following phase cycle was used φ_1_ = x, −x; φ_2_ = 8(x), 8(−x); φ_3_ = 2x, 2(−x), 2y, 2(−y), and ψ = x, 2(−x), x, y, 2(−y), y, (−x), 2x, (−x), (−y), 2y, (−y). For more details, see Section 2, Bruker pulse program included in Data S1

The acquisition of proton NMR spectra of high‐alcohol strength samples is severely hindered by the high concentrations of both water and ethanol. To overcome this issue, it is possible to dry the sample and redissolve it in a deuterated solvent^45^; however, this has obvious drawbacks in increased labour and loss of volatile compounds and thus working with original liquids is preferred. Excitation sculpting^17^ is a very efficient water suppression technique that has been adopted for the suppression of multiple signals.^46^ However, there are issues with this approach, as the intensity of a wider range of resonances is affected by applied selective inversion pulses. Employment of longer pulses, which partially addresses this issue, leads to unwanted *J*‐modulation of multiplets. The latter problem can be addressed by the perfect‐echo pulse sequence^19^ that nevertheless can induce intensity variations due to relaxation over the extended periods spins spend in the transverse plane. Methods that deal with the solvent suppression prior to spin excitation are therefore preferable. Water suppression enhanced through T_1_ effects or WET^47^, ^48^ is one such method. It has been recently successfully applied to acquire 1D and 2D spectra of strong distilled spirits produced from grape pomace.^49^ This study was performed using a room temperature probe, where WET performs well. Nevertheless, the efficiency of WET is known to deteriorate on cryoprobe instruments and a modification has been put forward that improves its performance.^50^ Another water suppression based method that suppresses solvent signals at the beginning of the pulse sequence is based on a 1D NOESY experiment.^51^ This technique is widely used in metabonomic studies and has also been adopted for multiple solvent suppression. Working with high alcohol strength spirits, Monokhova et al. presented an automated eightfold (the OH singlet, the ethanol triplet, and quartet) one‐channel signal suppression technique using a single combined shaped pulse.^52^ A similar method was implemented by Ragone et al. to examine wine samples.^53^ Described as line‐selective methods, they nevertheless do not achieve solvent suppression by irradiating selected lines of ethanol multiplets only. As demonstrated by Kupce et al., when shaped pulses targeting very close frequencies are combined in a single selective pulse, they do not work in a line‐selective manner; instead, they will saturate a broader region.^54^ We have therefore decided to abandon the “line‐selective” approach and focused on the multiplet‐selective solvent suppression instead within the framework of the 1D NOESY pulse sequence. In addition, we have also introduced suppression of ^13^C satellites that is compatible with cryoprobes. The proposed pulse sequence is shown in Figure 1.

Crucial to the quality of solvent suppression is the determination of the exact frequencies of solvent signals. However, due to the high Q factor of cryoprobes, even a very short r.f. pulse produces radiation damping,^55^ which severely distorts ethanol multiplets and makes this task impossible when focusing on ^12^C isotopomers of ethanol. This is not an issue for their much weaker ^13^C satellites signals, which can be acquired as the first trace of a nonrefocused gradient‐selected HSQC^56^ or nonrefocused reverse INEPT^57^ experiment. The former method produced distorted multiplets affected by the evolution of proton‐proton couplings, whereas the latter method yielded pure antiphase multiplets with respect to ^1^ *J*_CH_. Despite the lower sensitivity of the latter method, signal‐to‐noise >4,000 was routinely obtained, which is more than adequate for accurate frequency determination. The level of the suppression of the main signals (<25% of the height of the satellite signals) was also sufficient. The chemical shifts of ^12^C isotopomers of ethanol were therefore obtained on the basis of the frequency of ^13^C isotopomers and considering the proton ^13^C/^12^C isotope shift as detailed in Section 2.

**Figure 2:**
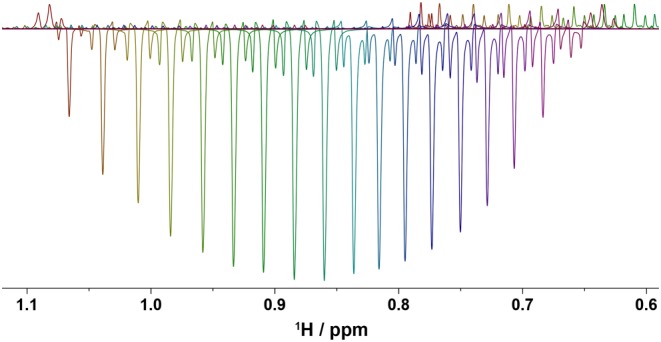
Residual signal of CH_3_ protons of ethanol obtained using the pulse sequence of Figure 1, as a function of the pulse length of the presaturation pulse. On the left, the pulse length, 50,094 μs, was calculated to allow 74*2π rotations, whereas the subsequent experiments increased this value by 38 μs. Each recalculated shape allowed for an additional phase shift increment of 20°. The range of 360° is shown in 17 increments. Note that the central line of the CH_3_ triplet is disproportionately taller

A double‐selective pulse of approximately 50 ms, saturating both CH_2_ and CH_3_ signals, was used to suppress ethanol signals. Its exact length was calculated for each sample taking into account the exact chemical shift difference between the CH_2_ and CH_3_ signals and allowing the off‐resonance signal (CH_3_ in this implementation) to make exactly a multiple of 2π rotation. We find that this is essential for excellent suppression of the off‐resonance signal as illustrated in Figure 2. Here, for a difference of 1,470 Hz between the CH_2_ and CH_3_ signals, a 50,094‐μs pulse produces 74*2π rotation of the off‐resonance CH_3_ signal (the first, left spectrum in Figure 2). When the pulse length was recalculated to allow additional precession in 20° increments the suppression of the CH_3_ signal became progressively worse. Lengthening the pulse by as little as 38 μs, or 0.076% of the original pulse length, which corresponds to the time needed to generate such as 20^o^ phase shift, has a noticeable effect.

The exact rotation of only one off‐resonance signal can be achieved in this way; therefore, another channel was used to suppress the water signal as described in Section 2.

Acquisition of ^13^C satellites can serve another purpose—quantification of the ethanol content of the sample. Indeed, calibrating the ethanol strength using water/ethanol mixtures based on integration of CH_2_ and CH_3_ signals for standard alcohol strengths in the region of 20% to 70% alcohol by volume (ABV) yielded a standard deviation of 4.5% ABV. This error was mostly due to baseline and phase distortions from the attenuation of the ^12^C isotopomer signal.

The next spectrum to be acquired was a 1D ^13^C NMR spectrum using inverse‐gated decoupling. This spectrum also serves a dual purpose. High ethanol concentrations yield single‐scan spectra with a signal‐to‐noise ratio > 740:1 at 43% ABV that are suitable for the quantification of the ethanol concentration. Indeed, this spectrum provides better quantification than the reverse INEPT, yielding a standard deviation for the same calibration of only 1.5% ABV. As the ^13^C signals display ethanol concentration‐dependent chemical shift changes and line broadening, these are peak picked and integrated using individually adjusted integral regions. The ^13^C chemical shift of the CH_2_ resonance varies up to 0.32 ppm (or 48 Hz at 150 MHz) between 20% and 70% ABV, shifting down field at higher alcohol strengths. The ^13^C CH_3_ resonance shifts by up to 0.20 ppm (or 30 Hz at 150 MHz) between 20% and 70% ABV, shifting up field at higher alcohol strengths. These chemical shift and line widths changes may be explained, in part, by the changing nature of water‐ethanol solutions at different ethanol concentrations, including hydrogen bond effects and ethanol aggregates.^58^, ^59^, ^60^

**Figure 3:**
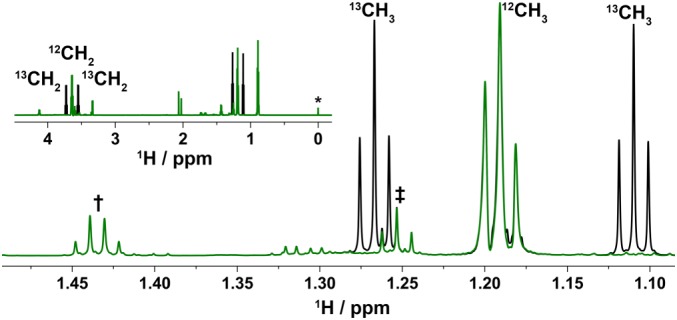
A partial ^1^H NMR spectrum of Scotch Whisky sample S15‐3896 acquired using the pulse sequence of Figure 1 with (green) and without (black) ^13^C ethanol isotopologue signals suppressed. This spectrum was acquired at 800 MHz. The ethanol CH_3_ region is shown in the main figure; the inset shows a broader range of the spectrum including DSS at 0 ppm (1 mM, indicated with *) and CH_2_ signals of ethanol at 3.64 ppm. Note that the signals of 3‐methylbutan‐1‐ol (1.43 ppm, indicated with †) overlay perfectly, indicating lack of any heating caused by the decoupling, and the triplet at 1.255 ppm (indicated with ‡) becomes clearly visible when the decoupling is activated

The major reason for acquiring 1D ^13^C NMR spectra is to determine the exact ^13^C chemical shifts of ethanol resonances. This information is required for setting up the parameters for the decoupling of ^13^C satellites of ethanol in the ^1^H spectrum of whisky. As it is not possible to apply broadband ^13^C‐decoupling during a 6‐s acquisition of ^1^H spectra on a cryoprobe instrument, decoupling of ethanol signals was performed in a selective manner. For this purpose, a cosine modulated rectangular pulse of approximately 2.5 ms was generated at the frequency midway through the two ethanol signals. As with the ^1^H presaturation pulse, its exact length was calculated to allow a 2π*n* rotation of both signals, as detailed in Section 2. This treatment resulted in a residual signal <1% of the original satellite signal height (Figure 3).

**Figure 4:**
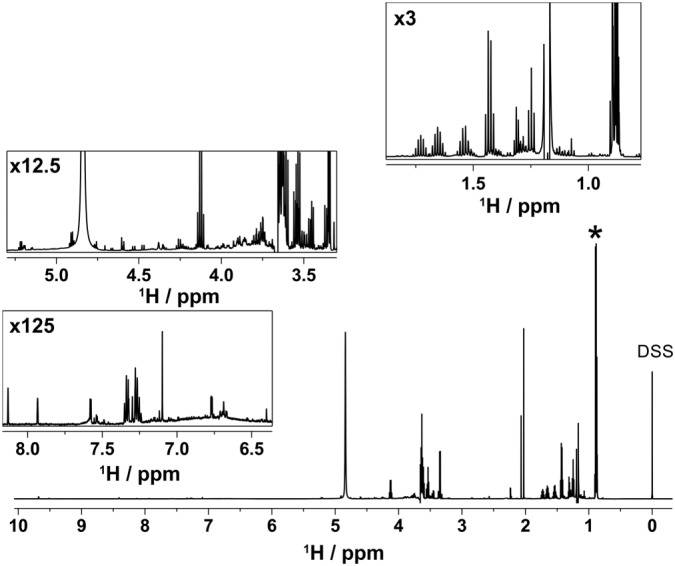
Typical ^1^H NMR spectrum of Scotch Whisky sample S14‐1941 with ethanol and water suppression, including the suppression of ^13^C satellite signals of ethanol acquired using the pulse sequence of Figure 1. Insets show three regions representing higher alcohols (0.8–1.8 ppm), carbohydrates (3–5.5 ppm) and cask extractives and aromatic compounds (6.5–8.5 ppm) indicating the vertical scale up relative to the main spectrum. The overlapped methyl signals from congeners at 0.88 ppm are labelled with an asterisk. DSS signals at 0 ppm act as a 1 mM internal standard

The final ^1^H NMR spectrum of Scotch Whisky shows remarkable suppression of water/ethanol signals with residual “solvent” signals smaller than the overlapping CH_3_ signals of higher alcohols at 0.88 ppm (Figure 4). The residual CH_2_ signals of ethanol are smaller than the signal of nine CH_3_ protons of 1 mM DSS used as internal standard. The level of the ethanol signals suppression can be quantified by recording a spectrum without the carbon decoupling (Figure 3). Here, the residual ^12^C isotopomer ethanol signals have approximately the same intensity as their satellites, representing suppression of 99.5% of the signal. The CH_2_ signals is typically better suppressed as its chemical shift coincides with the carrier frequency, whereas the CH_3_ is off‐resonance, contains three protons and displays a simpler multiplet. The water signal is typically suppressed to the level of the DSS signal.

The final spectrum shows many congeners clearly identifiable and quantifiable, displaying pure phase multiplets as shown in Figure 4. The signal‐to‐noise ratios for signals of several compounds are summarised in Table 1. Their identity was confirmed through the analysis of 2D experiments and spike‐in 1D spectra. We estimate that the limit of detection is therefore approximately 50 μM. This could be improved by increasing the number of scans or using a higher field instrument.

**Table 1:** Signal to noise ratios (SNRs) for resonances of several identified compounds in sample S14‐1941

Compound	δ ppm	Concentration (mM)		Multiplicity	# H	SNR
DSS‐d_6_	0.00	‐	1	s	9	14,623
3‐Methyl‐1‐butanol	1.43	6.8^a^	7.1	q	2	6,442
n‐Propanol	1.53	2.8^a^	3.5	m	2	1,589
Acetaldehyde	4.13	2.50^a^	2.50	q	2	465
Glucose	5.19/4.59	0.59^a^	0.40	s	2	117/270
Furfural	6.77	0.16^a^	0.13	dd	1	63
2‐Phenylethanol	7.34/7.27	0.21^a^	0.23	m	5	112/127
Syringaldehyde	7.3	0.059^a^	0.051	s	2	66

a Concentrations quoted were provided by the Scotch Whisky Research Institute

In conclusion, the presented protocol allows automated acquisition of high‐quality ^1^H NMR spectra of Scotch Whisky in 32 scans over 15 min on a 600‐MHz cryoprobe NMR spectrometer. The obtained spectra are suitable for chemometric analysis and quantitative analysis of congeners of Scotch Whisky or other spirits.

### 3.3 1D and 2D experiments with solvent suppression

To aid in interpretation of whisky spectra, several established NMR experiments were modified to include the developed solvent suppression scheme. Amongst these are homonuclear 1D and 2D experiments such as 2D COSY, 2D TOCSY, 2D band‐selective TOCSY, 1D chemical‐shift‐selective TOCSY, and 2D *J*‐resolved as well as heterocorrelated experiments including 2D ^1^H, ^13^C HSQC, 2D ^1^H, ^13^C HSQC‐TOCSY, and 2D ^1^H, ^13^C HMBC.

In the following, these experiments are briefly discussed in reference to the original experiments and illustrated using partial spectra of a 12‐year‐old Highland single malt Scotch Whisky (S14‐1941) and a 12‐year‐old Lowland single malt Scotch Whisky (S14‐1963). The spectra presented below were generated using appropriate apodization followed by Fourier transformation and baseline correction in both dimensions. Additional measures such as a *t*_1_ noise reduction produced cleaner spectra that are presented in Data S1 together with the full spectra.

## 4 HOMOCORRELATED EXPERIMENTS

**Figure 5:**
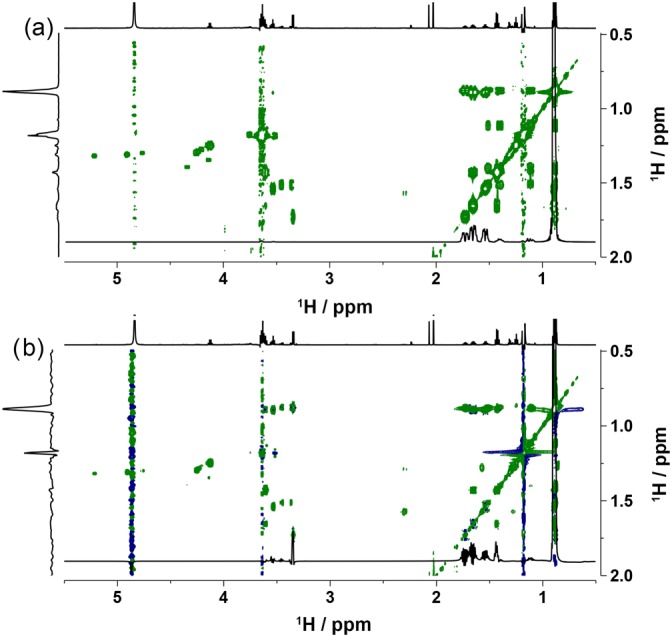
Partial (a) 2D COSY and (b) 40‐ms mixing time 2D TOCSY spectra of Scotch Whisky sample S14‐1941 acquired using the developed solvent suppression scheme of Figure 1. A 1D spectrum is shown at the top, scaled to show appropriate features, whereas vertical projections are shown on the sides. Horizontal traces taken at 0.88 ppm are scaled appropriately and overlaid to highlight spectral features

Establishing proton‐proton chemical shift correlations is essential for the structure elucidation of compounds and is typically achieved using *J*‐coupling constants to transfer the magnetisation in a COSY‐ or TOCSY‐type of experiments. The usefulness of through space correlations for small molecular weight congeners is limited, and NOESY experiments were therefore not included in this set. Acquisition of COSY spectra in aqueous solutions is challenging,^61^ and therefore, a basic gradient‐selected 2D COSY method (Bruker pulse sequence *cosygpppqf*) was used here. For acquisition of 2D TOCSY spectra, a phase‐sensitive method with DIPSI‐2^31^ and ZQ‐suppression scheme^33^ applied before and after the mixing time were chosen. Both methods yielded excellent results as seen in Figure 5. The level of the solvent suppression can be judged by inspecting vertical projections positioned at the sides of the 2D spectra. The methyl signals of higher alcohols resonating at 0.88 ppm are more intense that the residual signal of the methyl protons of ethanol. Several correlations between aliphatic protons resonating in a 1 ppm region (0.8–1.8 ppm) to the methyl protons around 0.88 ppm and other protons up to 5.2 ppm can be seen. These protons belong to higher alcohols, such as 2‐ and 3‐methylbutan‐1‐ol, n‐propanol, and iso‐butanol. An example of the full COSY spectrum acquired is provided in Figure S5 and with t_1_ noise digitally removed in Figure S6. The full TOCSY spectrum acquired is provided in Figure S7 and with t_1_ noise digitally removed in Figure S8.

**Figure 6:**
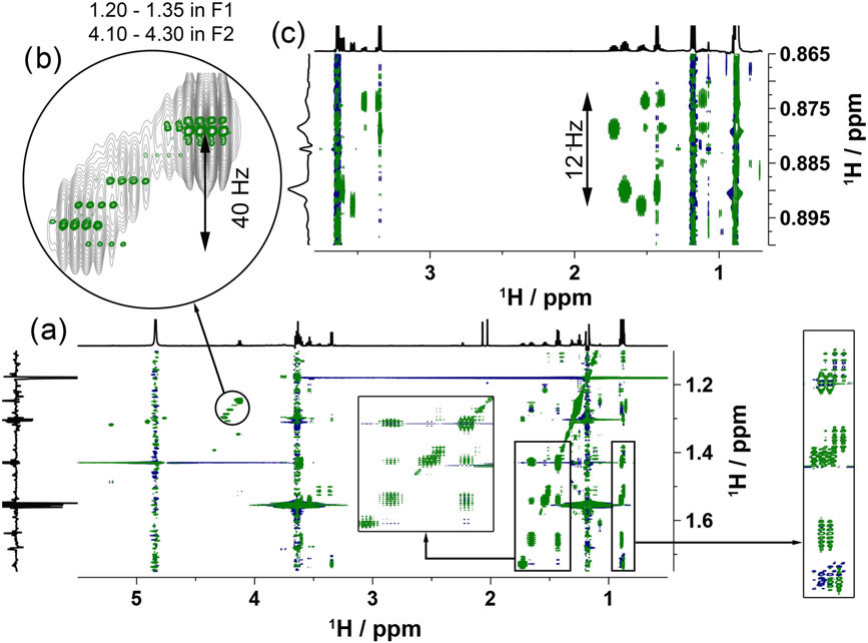
Partial band‐selective 2D TOCSY spectra of Scotch Whisky sample S14‐1941 acquired using the solvent suppression scheme of Figure 1 and 80‐ms mixing time. Separately, acquired 1D spectra are shown as external horizontal projections, whereas internal vertical projections are shown. (a) A region of 500 Hz centred on 1.43 ppm was inverted by a 10‐ms IBurp2 pulse. Two expansions are shown as indicated by arrows; (b) expansion of a circled region from (a) in green overlaid with the same region of 2D TOCSY spectrum shown in Figure 5b. (c) In a separate experiment, a region of 50 Hz centred on 0.88 ppm was inverted by a 20‐ms IBurp2 pulse

As with every complex mixture, compounds containing very similar fragments are present at different concentrations. This leads to strong signals concealing the weak ones and preventing their identification, especially when wide proton multiplets are present. Their decoupling in one dimension is therefore highly desirable, while preserving their multiplicity in the other, thus retaining important information provided by *J*‐couplings. Towards this end, we have implemented a solvent suppression scheme into a band‐selective 2D TOCSY experiment^32^ achieving excellent resolution as illustrated in Figure 6.

In the example presented in Figure 6a, a 500‐Hz region centred at 1.43 ppm was selectively inverted, which contains some mutually coupled protons, preventing refocusing of *J*‐couplings. Still, even this region benefits from excellent resolution afforded by a long *t*_1_ acquisition time (576 ms) as shown in the inset of Figure 6a. Protons from within this region resonating in a small window of 40 Hz centred on 1.13 ppm do not have coupled partners in the inverted region but are *J*‐coupled to protons resonating at around 4.05 ppm. Consequently, they appear as singlets in *F*_1_ as shown in Figure 6b. At least 10 resonances were identified in this narrow region due to removal of the couplings in *F*_1_. In a separate experiment, methyl signals at 0.88 ppm resonating within 15 Hz could also be resolved in *F*_1_ by applying a 20‐ms inversion pulse that does not invert any additional resonances. Several cross peaks in two regions around 1.6 and 3.4 ppm were clearly resolved connecting the protons resonating here with the methyl groups (Figure 6c).

**Figure 7:**
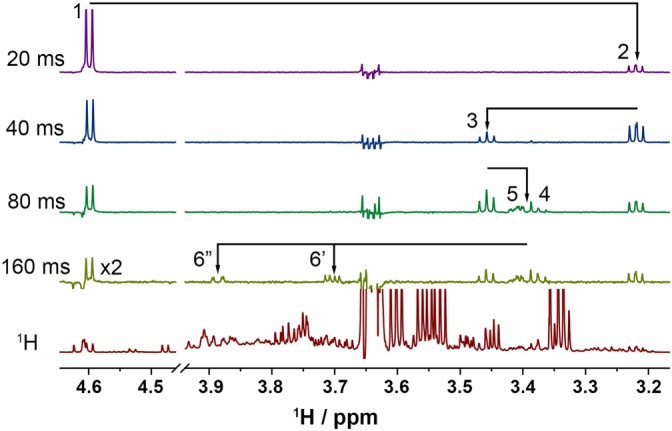
Selective 1D CSSF TOCSY of β‐d‐glucose in Scotch Whisky sample W16‐1009 with mixing times from 20 to 160 ms showing TOCSY‐transfer throughout the ring all the way to H‐6 protons. The 160‐ms mixing time spectrum had the y‐scale multiplied by a factor of 2. The residual signal at 3.64 ppm belongs to CH_2_ protons of ethanol. Spectra acquired at 800 MHz

Limited signal overlap in certain regions of ^1^H NMR spectra of Scotch Whisky allows the separation of signals in one‐dimensional experiments using highly selective chemical‐shift correlated experiment, CSSF 1D TOCSY.^34^ The original CSSF‐TOCSY pulse sequence was modified to include the solvent suppression element as described above. In an example given in Figure 7, the anomeric proton of β‐glucose at 4.52 ppm was selected and subjected to isotropic mixing of increasing length.

Note that a partially overlapping signal to the left of the H‐1 of β‐d‐glucose was not excited and the protons of the glucose ring were assigned in a few minutes. Also noticeable is a remarkable suppression of the CH_2_ signals of ethanol.

**Figure 8:**
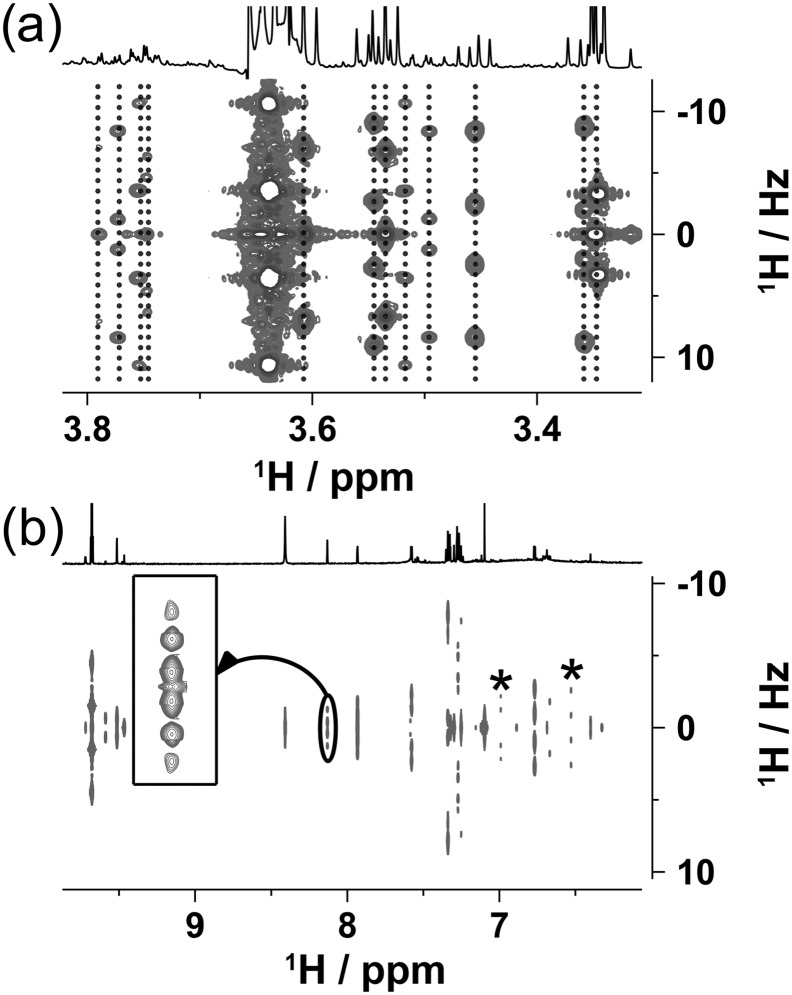
Magnitude mode 2D *J*‐resolved spectrum of Scotch Whisky sample S14‐1941 (a) region around the CH_2_ signal of ethanol (b) aromatic region. The spectrum was tilted and symmetrised in *F*_1_. Vertical dotted lines have been added to (a) to highlight the resolution in *F*_2_ obtained. The multiplets labelled with an asterisk in (b) belong to signals that are at the noise level in the 1D spectrum. The inset in (b) shows the multiplet of a signal at 8.13 ppm that is unresolved in the 1D spectrum

The last homonuclear experiment discussed is a 2D *J*‐resolved experiment. *J*‐resolved spectra have been used in the analysis of complex mixtures^62^, ^63^ due to their ability to resolve overlapping multiplets and expose the existence of very small coupling constants. Due to its simplicity, a magnitude mode *2D J*‐resolved experiment^64^ was selected and the developed signal suppression technique was implemented. High‐quality spectra were obtained revealing multiplets of numerous minor compounds. The solvent suppression was very efficient, as illustrated in Figure 8a, where a region around the CH_2_ resonances of ethanol is displayed.

Here, many minor signals are clearly resolved with only a 40‐Hz wide strip lost to the t_1_ noise of ethanol signal. Note the partially overlapping triplet to the right of the CH_2_ signal of ethanol that is well resolved in the *J*‐resolved spectrum. The aromatic region of the spectrum (Figure 8b) revealed valuable details of signals that were at the noise level in the 1D NMR spectrum. In addition, the magnetic field inhomogeneity compensating capacity of *J*‐resolved spectra uncovered splittings that are very valuable in establishing the identity of signals (see the inset in Figure 8b). An example of the full *J*‐resolved spectrum acquired is provided in Figure S9.

## 5 HETEROCORRELATED EXPERIMENTS

Reflecting the 1% natural abundance of ^13^C, heterocorrelated spectra of Scotch Whisky will only show the more abundant compounds present. This simplification, together with access to ^13^C chemical shifts and heteronuclear connectivity, makes these experiments very powerful, assisting significantly with unambiguous compound identification. The challenge associated with these experiments again is the solvent suppression, this time of both ^12^C and ^13^C isotopomers of ethanol. Insufficient suppression of the former can lead to excessive t_1_ ridges, whereas the failure to suppress the latter would produce very strong ethanol cross peaks obscuring the signals of interest.

**Figure 9:**
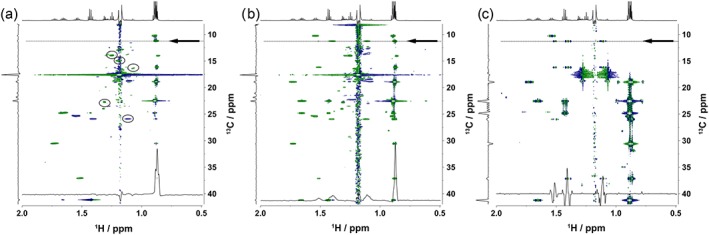
Partial 2D ^1^H, ^13^C correlated spectra of Scotch Whisky S14‐1941 (a) HSQC, (b) HSQC‐TOCSY, and (c) HMBC showing the low ppm aliphatic region. The 1D spectra are shown at the top, whereas projections are presented on the left‐hand side of the spectra. Signals close to CH_3_ protons of ethanol in both the ^12^C and ^13^C isotopomers are circled in the HSQC spectrum. Although the residual CH_2_ cross peak of ethanol is a singlet in (a) and (b), it appears as a ^1^ *J*_CH_ doublet in (c) as ^13^C decoupling is not applied here. Rows taken at δ (^13^C) = 11.22 ppm highlighted by arrows are presented at the bottom of the spectra

When implemented into standard Bruker pulse programs (see Section 2), the proposed solvent suppression produced excellent results. No additional measures were required for the HSQC and HSQC‐TOCSY experiments, whereas additional solvent suppression was implemented into the HMBC pulse sequence. Partial spectra for all three methods displaying the aliphatic region are shown in Figure 9. Full spectra for these experiments are shown in Figures S10 (HSQC), S11 (HSQC with t_1_ digitally removed), S12 (HSQC‐TOCSY), S13 (HSQC‐TOCSY with t_1_ digitally removed), S14 (HMBC), and S15 (HMBC with t_1_ digitally removed).

In all cases, the *t*_1_ noise is below the intensity of most of the cross peaks signals. The rudimentary decoupling of satellite signals reduced the ethanol cross peaks dramatically; nevertheless, these still are the most intense signals in the HSQC and HSQC‐TOCSY spectra. At the same time, this method allowed for the observation of signals that are very close, or even overlap, with the signals of ^12^C and ^13^C isotopomers of the protons of ethanol. The 2D CT‐HMBC required minor modification to improve the solvent suppression due to the relaxation effects during the long‐range proton‐carbon coupling evolution interval. During this interval, which could be up to 100 ms, a CW presaturation of water and CH_2_ signals at low power was applied improving their suppression.

## 6 CONCLUSIONS

We have presented a simple and robust solvent suppression technique that enables acquisition of high‐quality NMR spectra of alcoholic beverages on cryoprobe instruments and applied it to acquire 1D and 2D NMR spectra of Scotch Whisky. The method uses three channels to suppress signals of water and ethanol, including those of ^13^C satellites of ethanol. It is based around the well‐established 1D NOESY solvent suppression technique and suppresses the solvent at the beginning of the pulse sequence, producing pure phase signals minimally affected by the relaxation. The acquisition of 1D ^1^H spectra is executed in automation allowing high throughput characterisation of Scotch Whisky. The developed solvent suppression was used to modify several homocorrelated and heterocorrelated 2D NMR experiments that provide a wealth of information and will assist in the structure elucidation of Scotch Whisky compounds, and generally the characterisation of alcoholic beverages in their native form. Example NMR spectra for the experiments outlined in this work are available online at https://doi.org/10.7488/ds/2064.

## Supporting information

Data S1
Figure 1 – 1D ^1^H NMR spectrum of Scotch Whisky with only ‐OH signal suppressed
Figure 2 – 1D ^1^H Reverse INEPT NMR spectrum of Scotch Whisky showing antiphase multiplets of ^13^C isotopomers of ethanol
Figure 3 ‐ 1D ^13^C NMR spectrum of Scotch Whisky showing singlets for ethanol
Figure 4 – 1D ^1^H NMR spectrum of Scotch Whisky with water and ethanol signals suppressed
Figure 5 ‐ 2D ^1^H, ^1^H COSY NMR spectrum of Scotch Whisky
Figure 6 ‐ 2D ^1^H, ^1^H COSY NMR spectrum of Scotch Whisky with t_1_ noise digitally removed using MestreNova 11
Figure 7 ‐ 2D ^1^H, ^1^H TOCSY NMR spectrum of Scotch Whisky
Figure 8 ‐ 2D ^1^H, ^1^H TOCSY NMR spectrum of Scotch Whisky with t_1_ noise digitally removed using MestreNova 11
Figure 9 ‐ 2D ^1^H, ^1^H *J*‐Resolved NMR spectrum of Scotch Whisky
Figure 10 ‐ 2D ^1^H, ^13^C HSQC NMR spectrum of Scotch Whisky
Figure 11 ‐ 2D ^1^H, ^13^C HSQC NMR spectrum of Scotch Whisky with t_1_ noise digitally removed using MestreNova 11
Figure 12 ‐ 2D ^1^H, ^13^C HSQC‐TOCSY NMR spectrum of Scotch Whisky
Figure 13 ‐ 2D ^1^H, ^13^C HSQC‐TOCSY NMR spectrum of Scotch Whisky with t_1_ noise digitally removed using MestreNova 11
Figure 14 ‐ 2D ^1^H, ^13^C HMBC NMR spectrum of Scotch Whisky
Figure 15 ‐ 2D ^1^H, ^13^C HMBC NMR spectrum of Scotch Whisky with t_1_ noise digitally removed using MestreNova 11
