# Supplementary material for: Advanced solvent signal suppression for the acquisition of 1D and 2D NMR spectra of Scotch Whisky
Source: Magn Reson Chem. 2017 Jun 29;55(9):785–96. doi: 10.1002/mrc.4621 (PMC5599976; doi:10.1002/mrc.4621)
Supplement: Supplementary file 1 — Data S1 Figure 1 – 1D 1H NMR spectrum of Scotch Whisky with only ‐OH signal suppressed Figure 2 – 1D 1H Reverse INEPT NMR spectrum of Scotch Whisky showing antiphase multiplets of 13C isotopomers of ethanol Figure 3 ‐ 1D 13C NMR spectrum of Scotch Whisky showing singlets for ethanol Figure 4 – 1D 1H NMR spectrum of Scotch Whisky with water and ethanol signals suppressed Figure 5 ‐ 2D 1H, 1H COSY NMR spectrum of Scotch Whisky Figure 6 ‐ 2D 1H, 1H COSY NMR spectrum of Scotch Whisky with t1 noise digitally removed using MestreNova 11 Figure 7 ‐ 2D 1H, 1H TOCSY NMR spectrum of Scotch Whisky Figure 8 ‐ 2D 1H, 1H TOCSY NMR spectrum of Scotch Whisky with t1 noise digitally removed using MestreNova 11 Figure 9 ‐ 2D 1H, 1H J‐Resolved NMR spectrum of Scotch Whisky Figure 10 ‐ 2D 1H, 13C HSQC NMR spectrum of Scotch Whisky Figure 11 ‐ 2D 1H, 13C HSQC NMR spectrum of Scotch Whisky with t1 noise digitally removed using MestreNova 11 Figure 12 ‐ 2D 1H, 13C HSQC‐TOCSY NMR spectrum of Scotch Whisky Figure 13 ‐ 2D 1H, 13C HSQC‐TOCSY NMR spectrum of Scotch Whisky with t1 noise digitally removed using MestreNova 11 Figure 14 ‐ 2D 1H, 13C HMBC NMR spectrum of Scotch Whisky Figure 15 ‐ 2D 1H, 13C HMBC NMR spectrum of Scotch Whisky with t1 noise digitally removed using MestreNova 11 [file MRC-55-785-s001.docx]

Advanced Solvent Signal Suppression for the Acquisition of 1D and 2D NMR Spectra of Scotch Whisky

Supplementary Information

Will Kew^a^, Nicholle G. A. Bell^a^, Ian Goodall^b^, Dušan Uhrín^a‡^

*^a^EastCHEM School of Chemistry, University of Edinburgh, King's Buildings, David Brewster Road, Edinburgh, EH9 3FJ, UK.*

^b^*The Scotch Whisky Research Institute, The Robertson Trust Building, Research Avenue*

*North, Riccarton, Edinburgh, UK, EH14 4AP*

^‡^*E-mail:*[*dusan.uhrin@ed.ac.uk*](mailto:dusan.uhrin@ed.ac.uk)

Keywords: NMR, ^1^H, ^13^C, complex mixture, Scotch whisky, solvent suppression, alcoholic beverages

Short Title: Signal Suppression for the Acquisition of Scotch Whisky NMR Spectra

# Table of Content

Pulse sequence for the acquisition of 1D ^1^H NMR spectra of whisky with solvent suppression. 3

[Figure 1 – 1D ^1^H NMR spectrum of Scotch Whisky with only -OH signal suppressed 6](#_Toc480273629)

[Figure 2 – 1D ^1^H Reverse INEPT NMR spectrum of Scotch Whisky showing antiphase multiplets of ^13^C isotopomers of ethanol 7](#_Toc480273630)

[Figure 3 - 1D ^13^C NMR spectrum of Scotch Whisky showing singlets for ethanol 8](#_Toc480273631)

[Figure 4 – 1D ^1^H NMR spectrum of Scotch Whisky with water and ethanol signals suppressed 9](#_Toc480273632)

[Figure 5 - 2D ^1^H, ^1^H COSY NMR spectrum of Scotch Whisky 10](#_Toc480273633)

[Figure 6 - 2D ^1^H, ^1^H COSY NMR spectrum of Scotch Whisky with t_1_ noise digitally removed using MestreNova 11 11](#_Toc480273634)

[Figure 7 - 2D ^1^H, ^1^H TOCSY NMR spectrum of Scotch Whisky 12](#_Toc480273635)

[Figure 8 - 2D ^1^H, ^1^H TOCSY NMR spectrum of Scotch Whisky with t_1_ noise digitally removed using MestreNova 11 13](#_Toc480273636)

[Figure 9 - 2D ^1^H, ^1^H J-Resolved NMR spectrum of Scotch Whisky 14](#_Toc480273637)

[Figure 10 - 2D ^1^H, ^13^C HSQC NMR spectrum of Scotch Whisky 15](#_Toc480273638)

[Figure 11 - 2D ^1^H, ^13^C HSQC NMR spectrum of Scotch Whisky with t1 noise digitally removed using MestreNova 11 16](#_Toc480273639)

[Figure 12 - 2D ^1^H, ^13^C HSQC-TOCSY NMR spectrum of Scotch Whisky 17](#_Toc480273640)

[Figure 13 - 2D ^1^H, ^13^C HSQC-TOCSY NMR spectrum of Scotch Whisky with t_1_ noise digitally removed using MestreNova 11 18](#_Toc480273641)

[Figure 14 - 2D ^1^H, ^13^C HMBC NMR spectrum of Scotch Whisky 19](#_Toc480273642)

[Figure 15 - 2D ^1^H, ^13^C HMBC NMR spectrum of Scotch Whisky with t_1_ noise digitally removed using MestreNova 11 20](#_Toc480273643)

For acquisition parameters, see main paper Materials and Methods.

# Pulse Sequence for acquisition of 1D 1H NMR spectra of whisky with solvent suppression

;whisky.du

;avance-version (11/01/17)

;1D with presaturation during relaxation delay and mixing time

; and spoil gradient

;using shaped pulse for multiple solvent presaturation

; (main solvent peak on resonance) during relaxation delay

;using cw presaturation during mixing time

;

;$CLASS=HighRes

;$DIM=1D

;$TYPE=

;$SUBTYPE=

;$COMMENT=

#include <Avance.incl>

#include <Grad.incl>

#include <Delay.incl>

"d12=20u"

"d13=4u"

"p2=2*p1"

"p17=700"

"p30=192"

"DELTA2=d2-2*p3-2u"

"if (d1/p23 < 1) {l6=1;} else {l6=d1/(p23);}"

"acqt0=-p0*2/3.1416"

1 ze

d12 pl22:f3

d12 pl9:f1

d12 pl24:f2

2 30m

d13 do:f2

# ifdef FLAG_BLK

4u BLKGRAD

# else

4u

# endif /*FLAG_BLK*/

# ifdef C13_DEC

d12 cw:f3 ph29

d12 cpds2:f2

3 (p23:sp23 ph29):f1

4u

lo to 3 times l6

d13 do:f3

d13 do:f2

# else

d12 cw:f3 ph29

3 (p23:sp23 ph29):f1

4u

lo to 3 times l6

d13 do:f3

# endif

50u UNBLKGRAD

p19:gp1

d16 pl1:f1

p1 ph1

4u

p1 ph2

4u

p16:gp2

d16

# ifdef FLAG_BLK

4u

# else

4u BLKGRAD

# endif /*FLAG_BLK*/

p0 ph3 ;READ PULSE

go=2 ph31

30m mc #0 to 2 F0(zd)

d13 do:f2

# ifdef FLAG_BLK

4u BLKGRAD

# else

4u

# endif /*FLAG_BLK*/

exit

ph1=0 2

ph2=0 0 0 0 0 0 0 0 2 2 2 2 2 2 2 2

ph3=0 0 2 2 1 1 3 3

ph27=16{0} 16{2}

ph29=32{0} 32{2}

ph31=0 2 2 0 1 3 3 1 2 0 0 2 3 1 1 3

;pl1 : f1 channel - power level for pulse (default)

;pl9 : f1 channel - power level for presaturation

;sp6: f1 channel - shaped pulse for presaturation

;p0 : for any flip angle

;p1 : f1 channel - 90 degree high power pulse

;p16: homospoil/gradient pulse

;p23: f1 channel - presaturation using shaped pulse

;d1 : relaxation delay; 1-5 * T1

;d12: delay for power switching [20 usec]

;d16: delay for homospoil/gradient recovery

;l6: p23 * l6 = total duration of presaturation

;NS: 8 * n, total number of scans: NS * TD0

;DS: 4

;for z-only gradients:

;gpz1: 50%

;gpz2: -11%

;use gradient files:

;gpnam1: SMSQ10.100

;gpnam2: SMSQ10.100


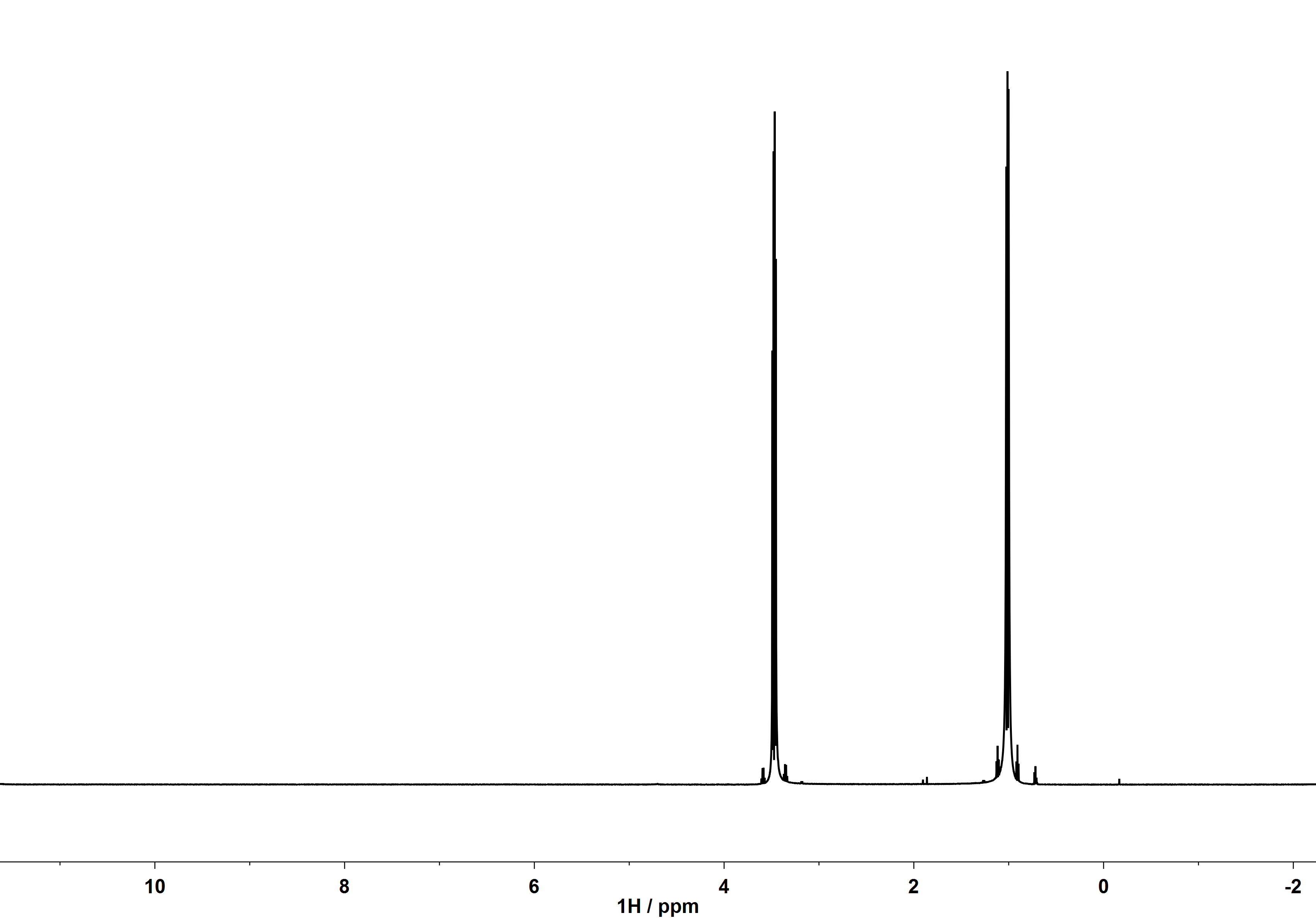


Figure 1 – 1D ^1^H NMR spectrum of Scotch Whisky with only -OH signal suppressed


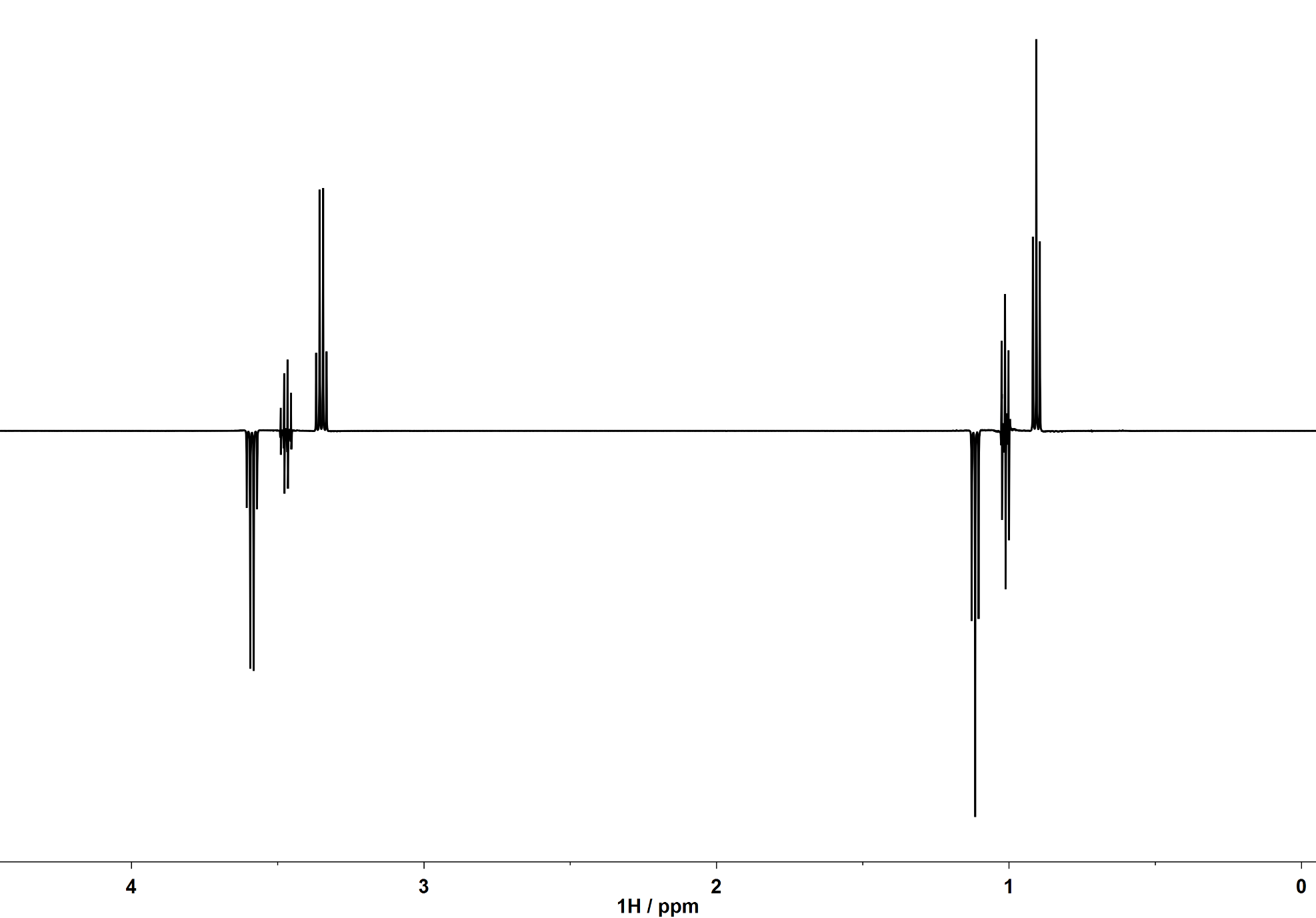


Figure 2 – 1D ^1^H Reverse INEPT NMR spectrum of Scotch Whisky showing antiphase multiplets of ^13^C isotopomers of ethanol


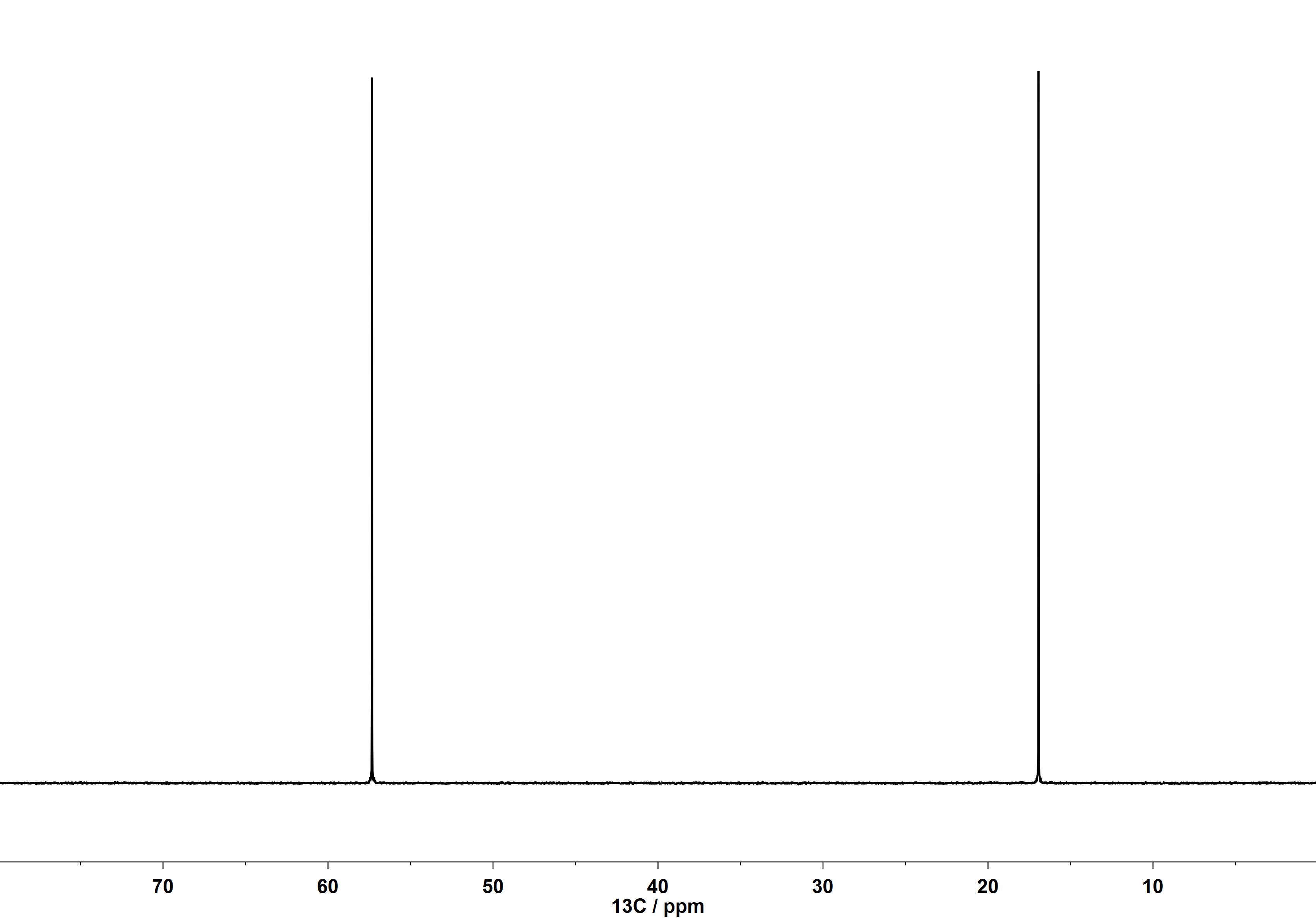


Figure 3 - 1D ^13^C NMR spectrum of Scotch Whisky showing singlets for ethanol


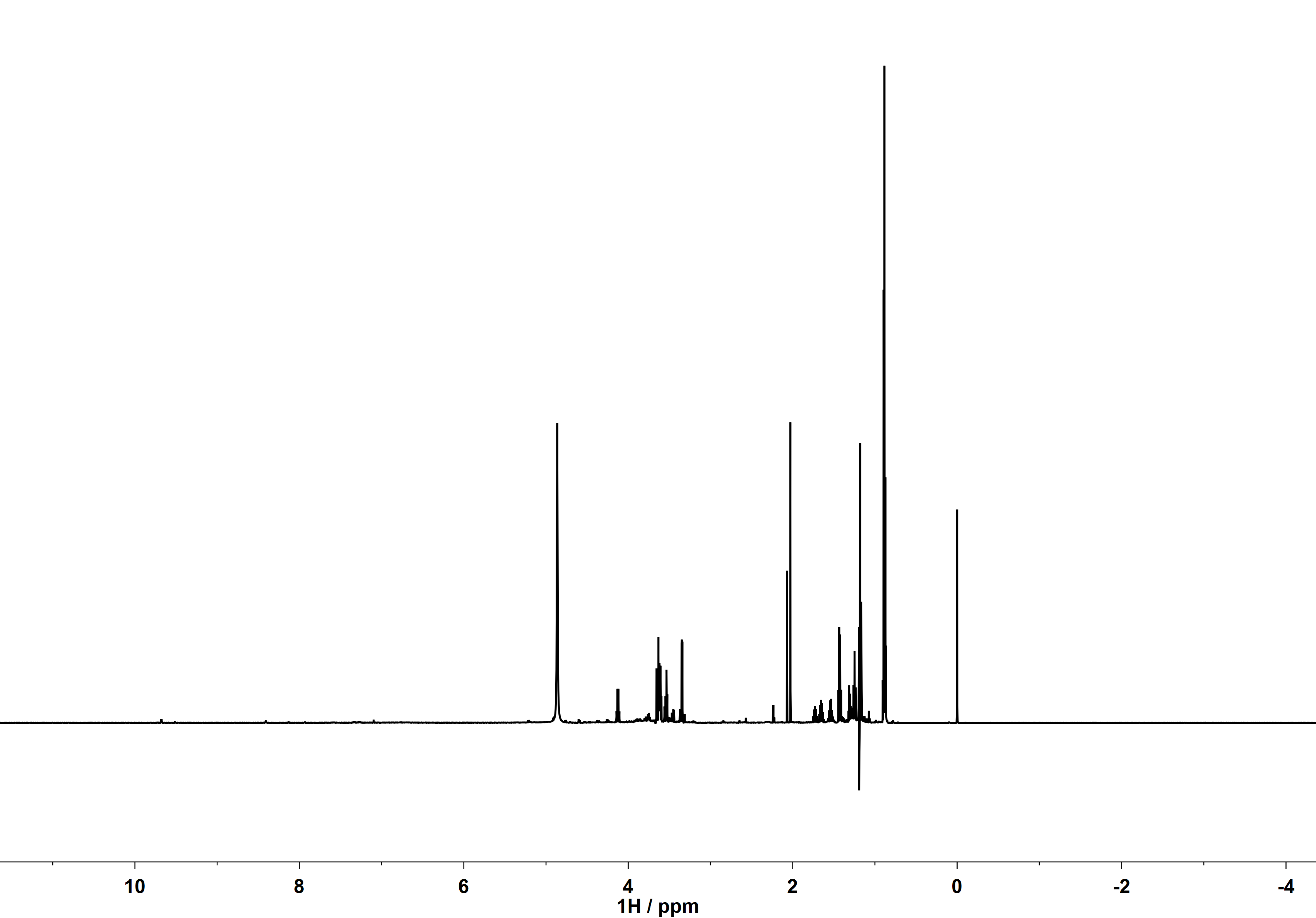


Figure 4 – 1D ^1^H NMR spectrum of Scotch Whisky with water and ethanol signals suppressed


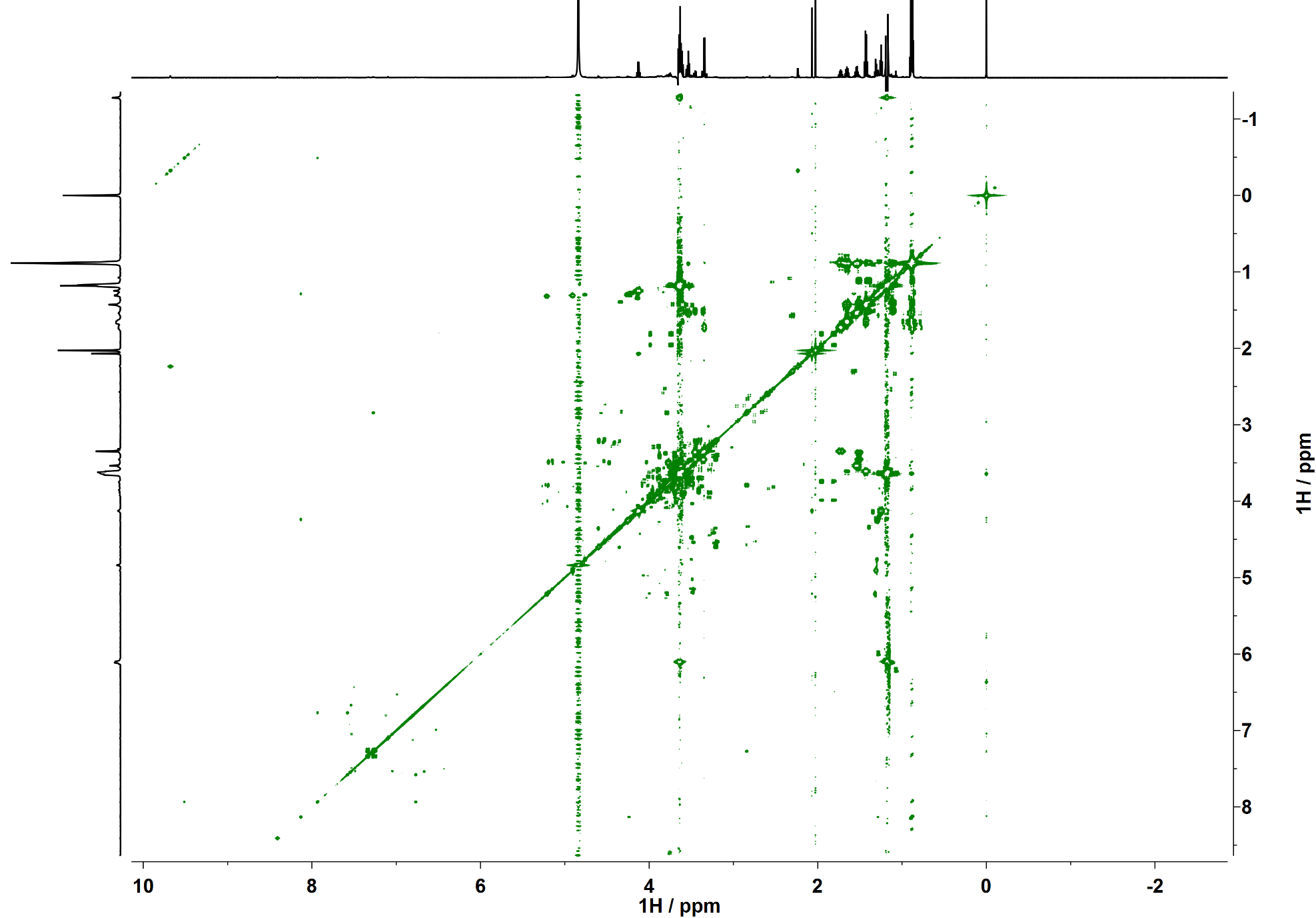


Figure 5 - 2D ^1^H, ^1^H COSY NMR spectrum of Scotch Whisky


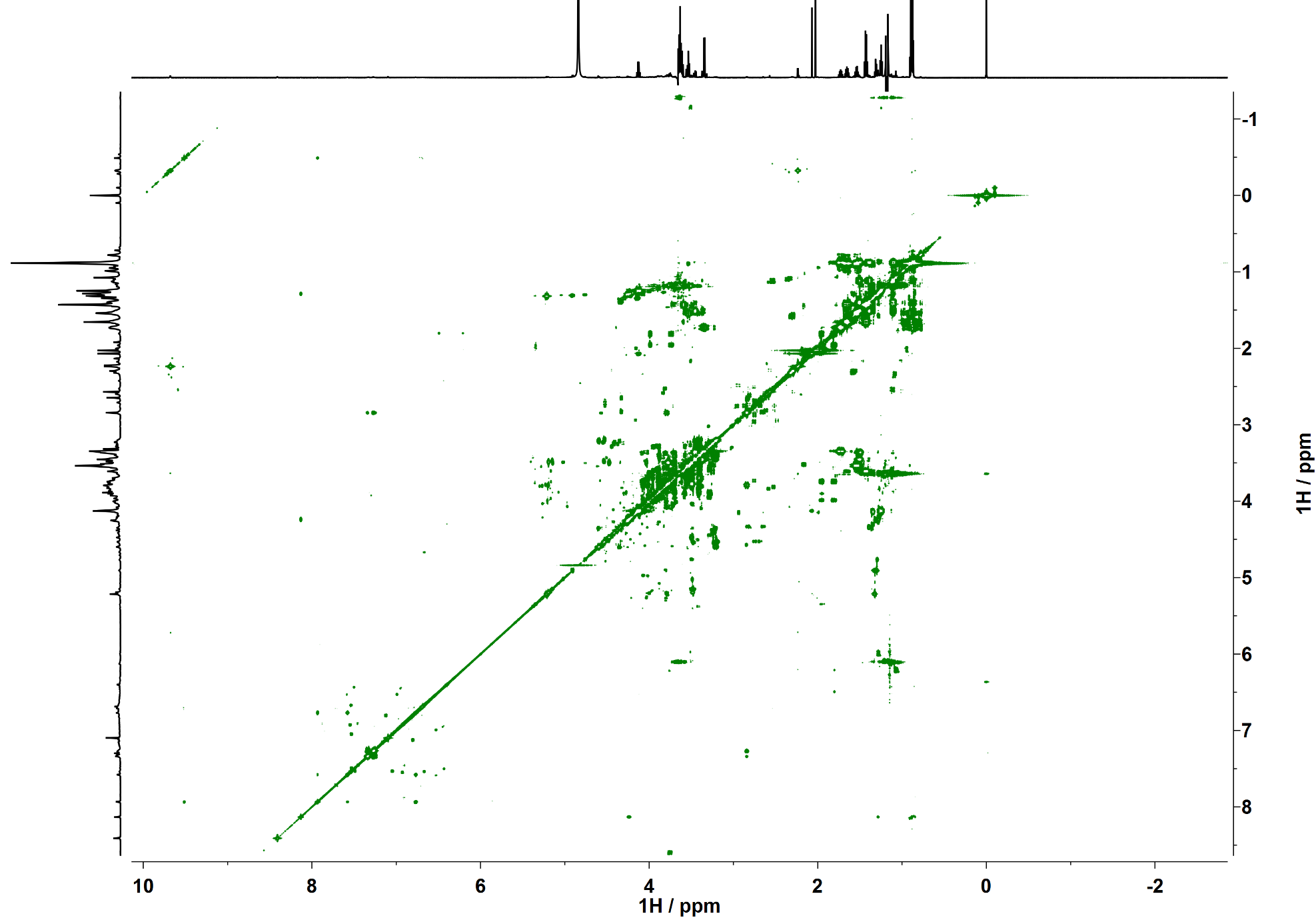


Figure 6 - 2D ^1^H, ^1^H COSY NMR spectrum of Scotch Whisky with t_1_ noise digitally removed using MestreNova 11


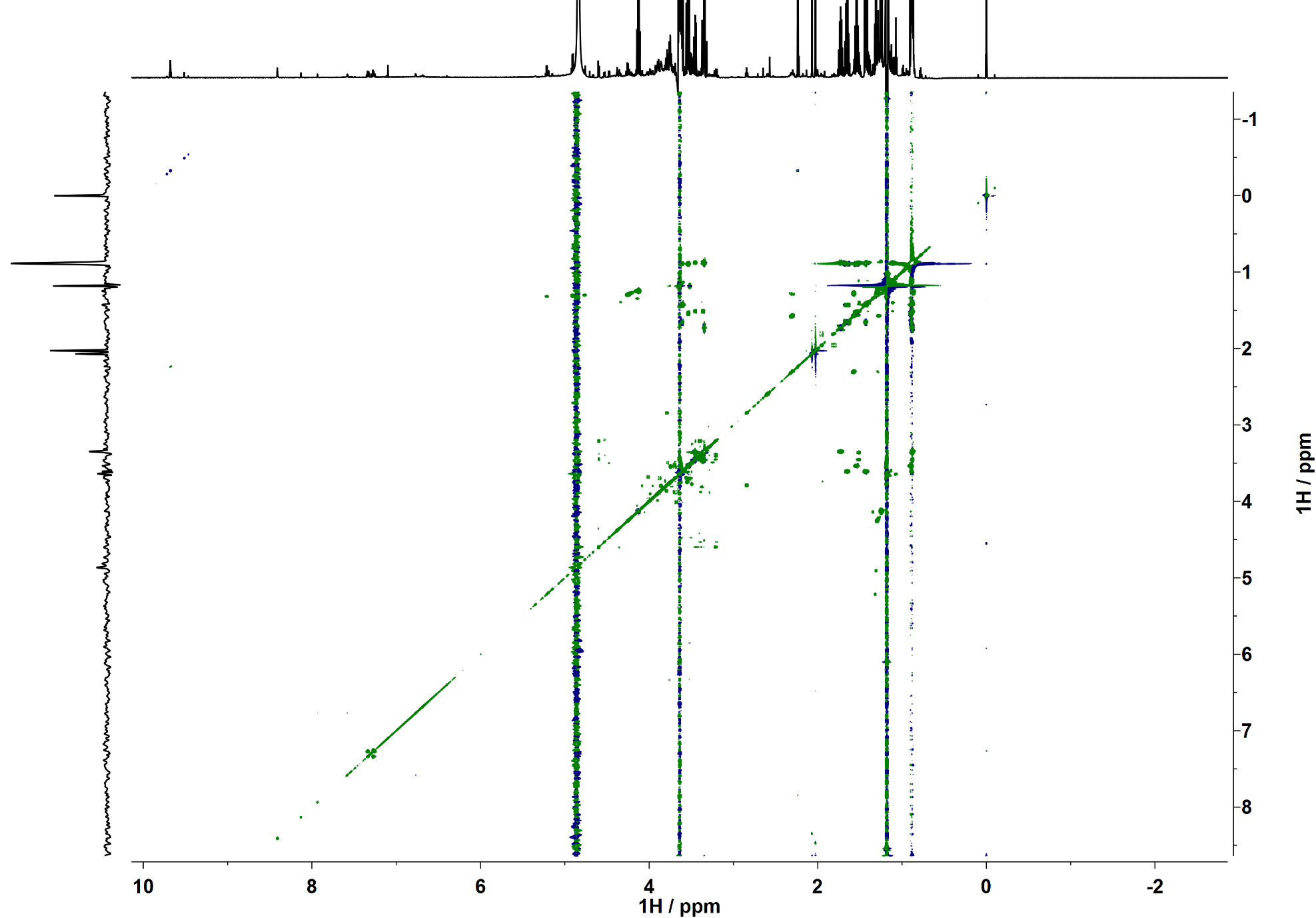


Figure 7 - 2D ^1^H, ^1^H TOCSY NMR spectrum of Scotch Whisky


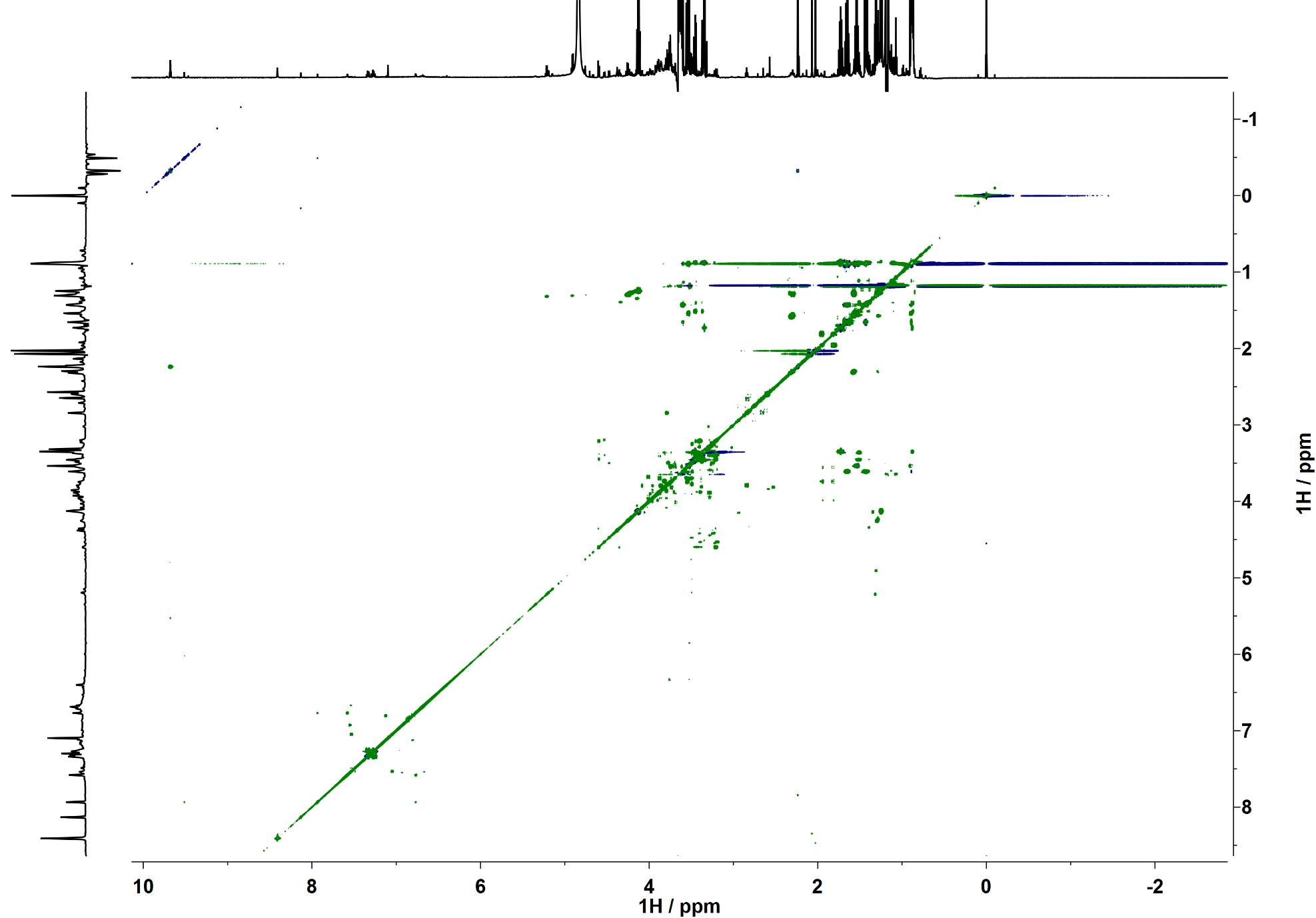


Figure 8 - 2D ^1^H, ^1^H TOCSY NMR spectrum of Scotch Whisky with t_1_ noise digitally removed using MestreNova 11


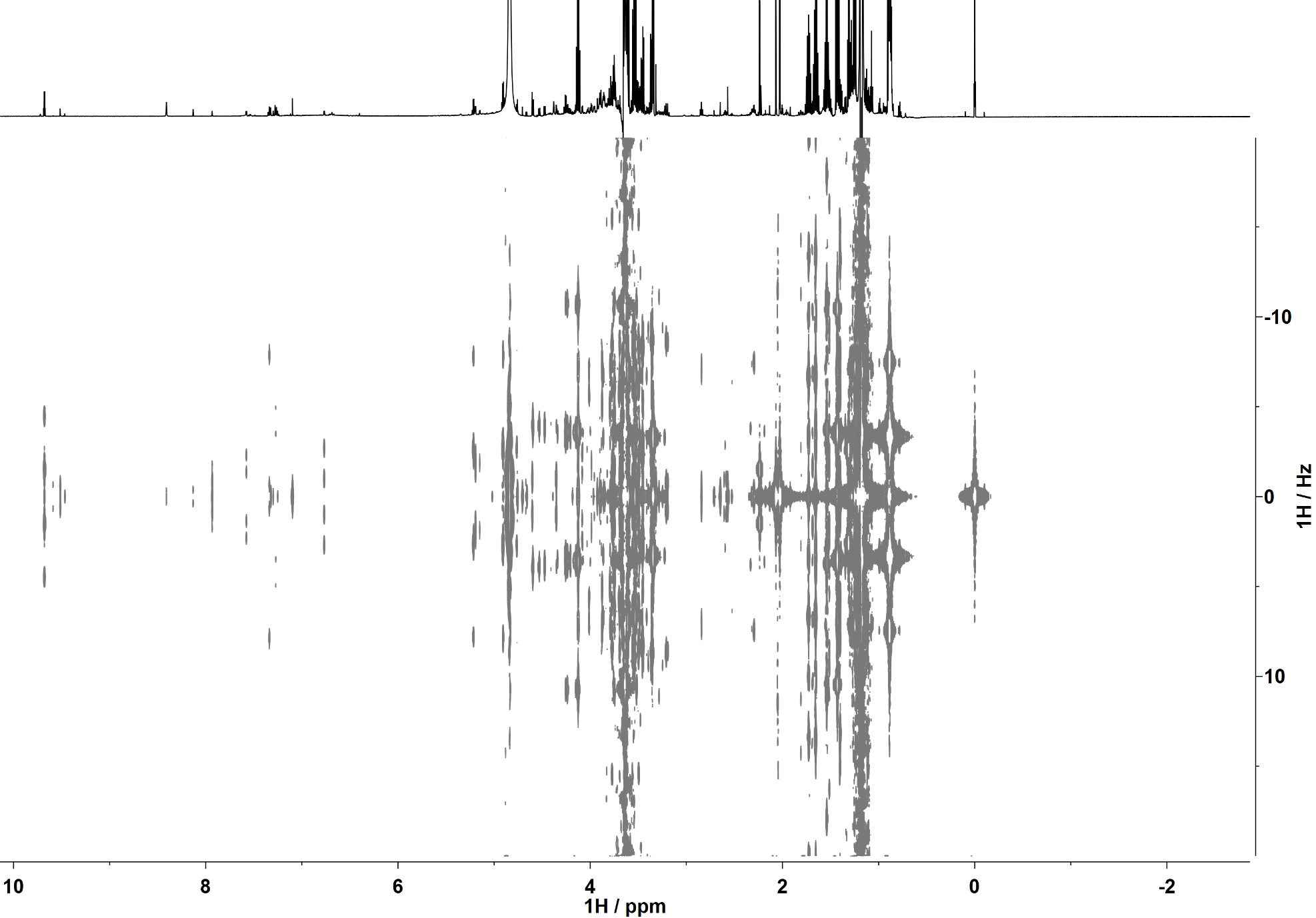


Figure 9 - 2D ^1^H, ^1^H J-Resolved NMR spectrum of Scotch Whisky


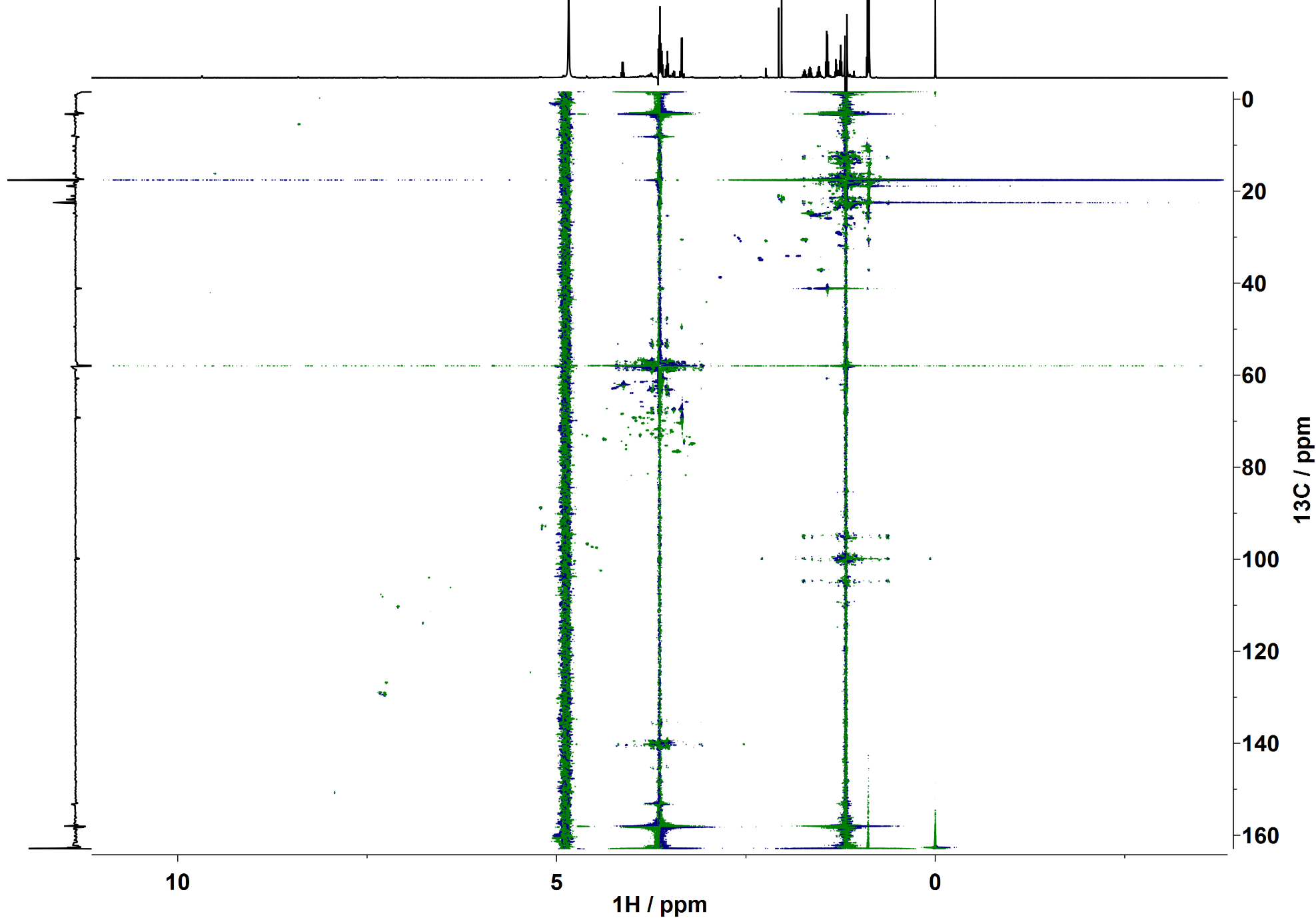


Figure 10 - 2D ^1^H, ^13^C HSQC NMR spectrum of Scotch Whisky


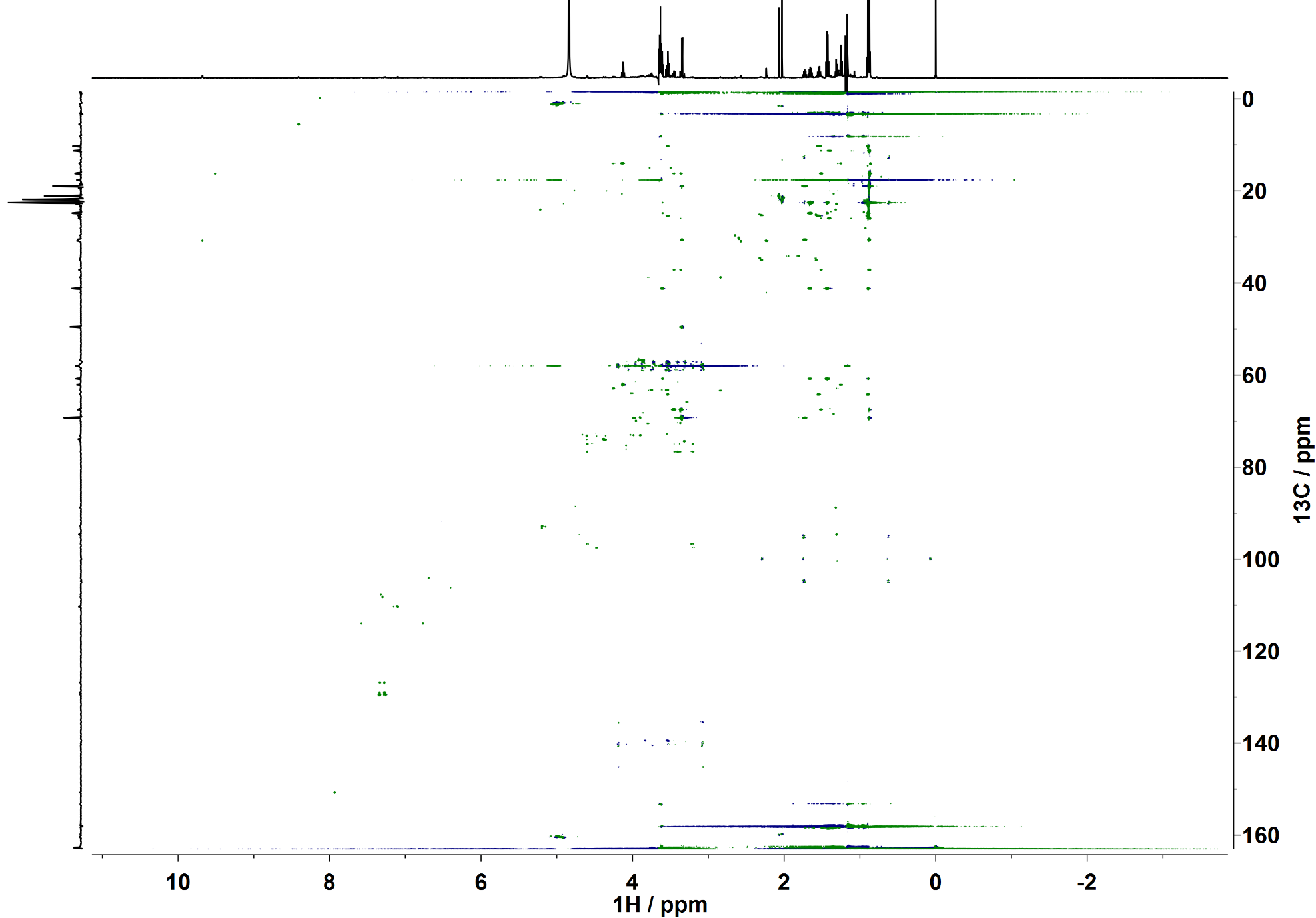


Figure 11 - 2D ^1^H, ^13^C HSQC NMR spectrum of Scotch Whisky with t1 noise digitally removed using MestreNova 11


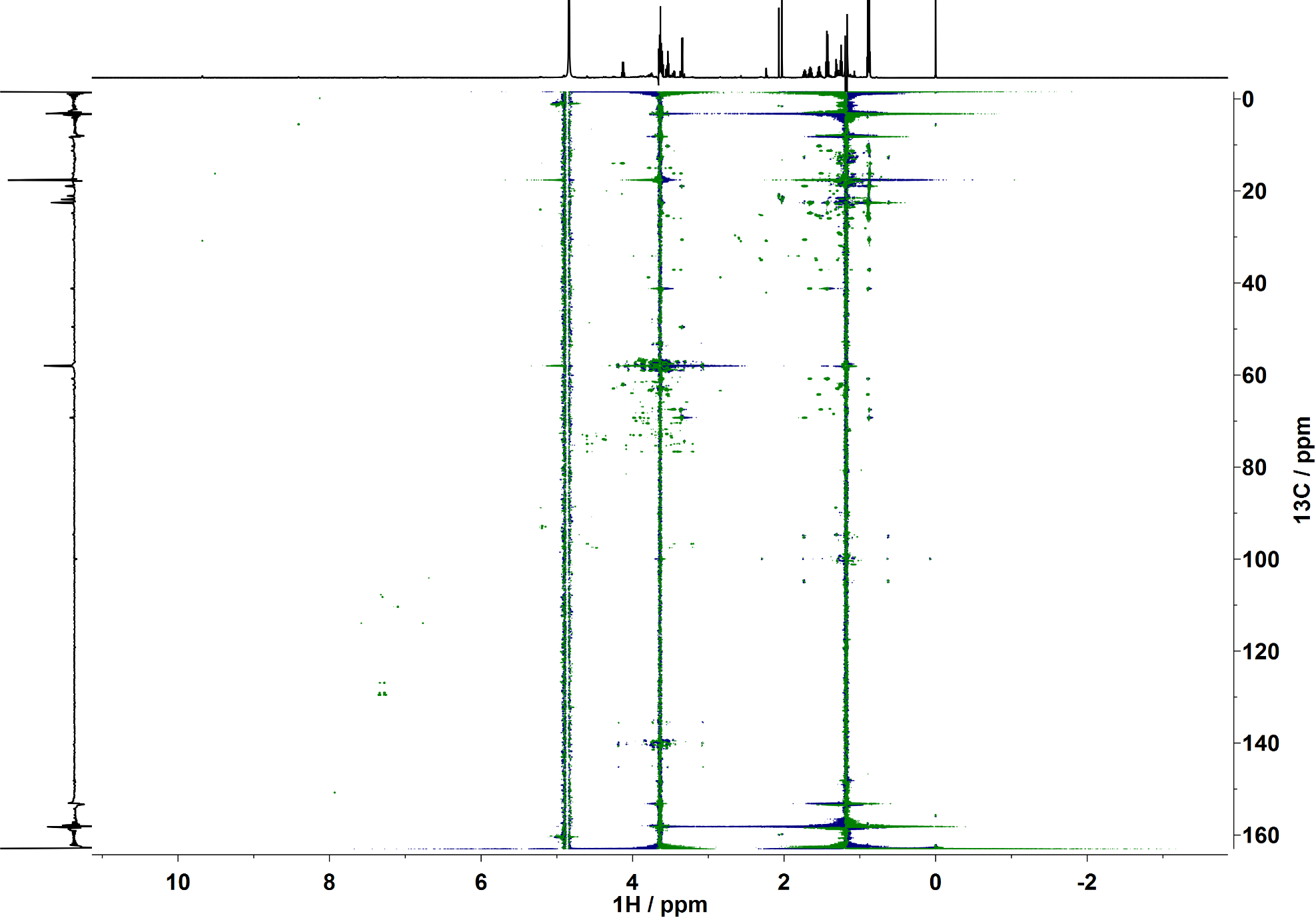


Figure 12 - 2D ^1^H, ^13^C HSQC-TOCSY NMR spectrum of Scotch Whisky


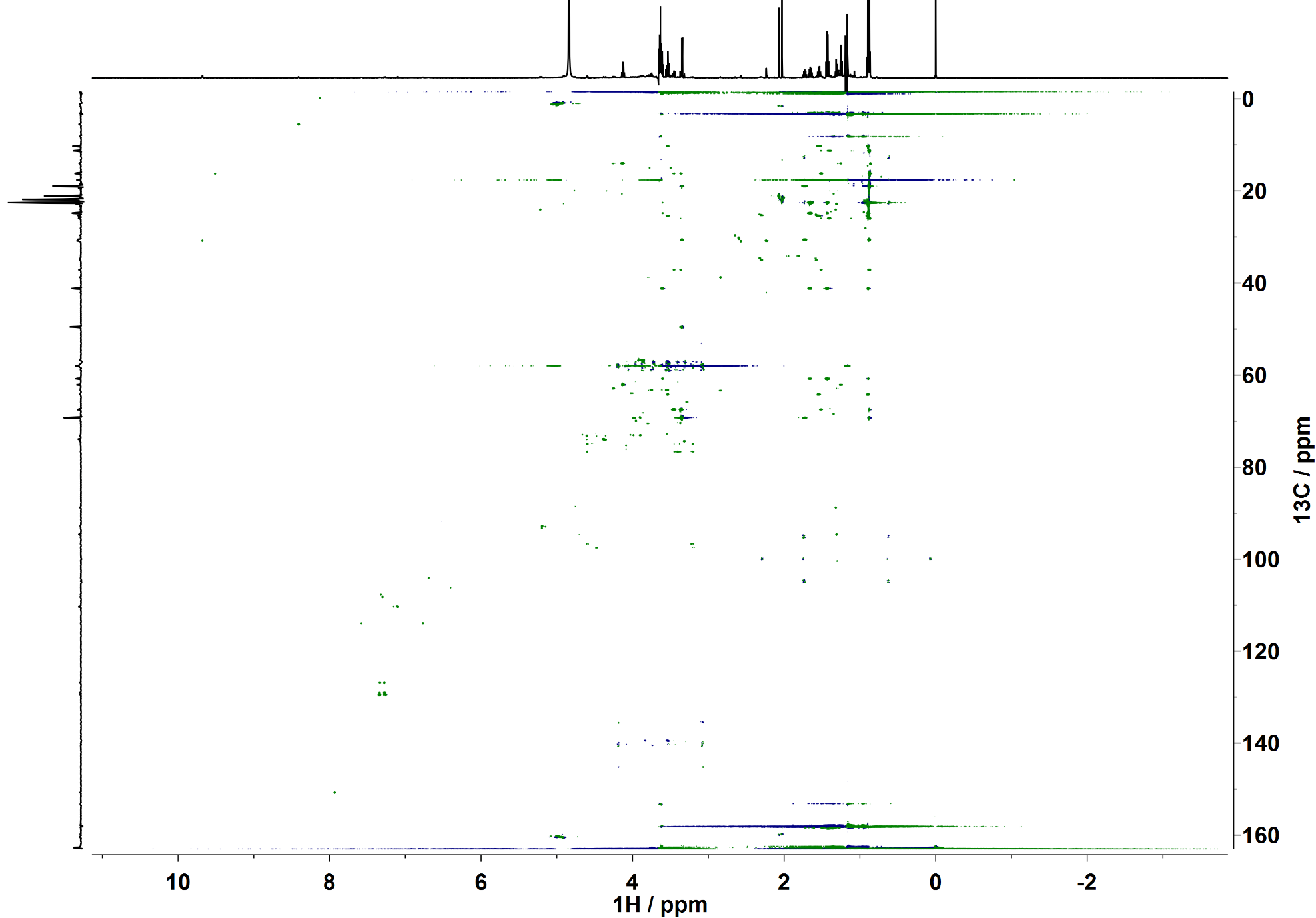


Figure 13 - 2D ^1^H, ^13^C HSQC-TOCSY NMR spectrum of Scotch Whisky with t_1_ noise digitally removed using MestreNova 11


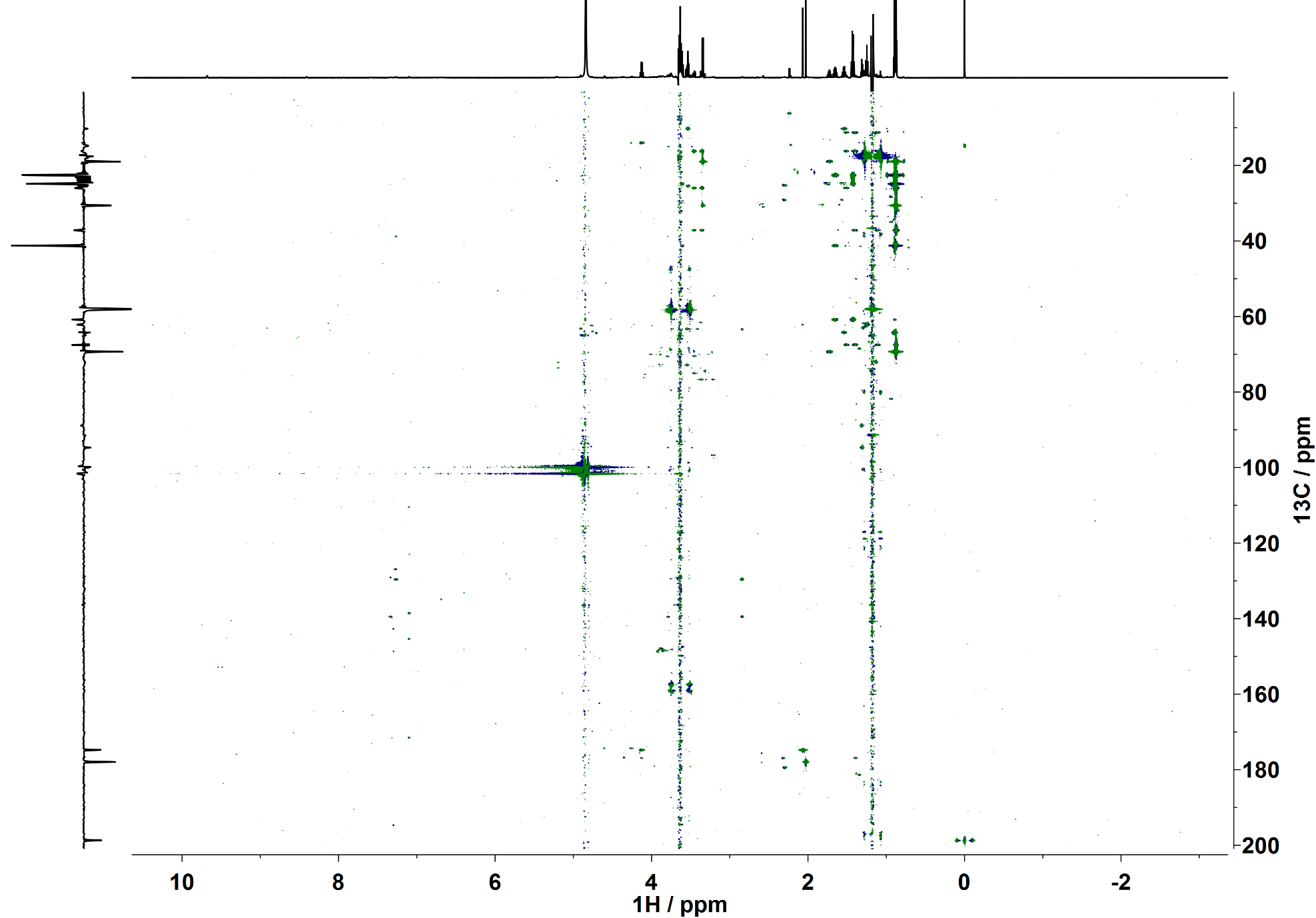


Figure 14 - 2D ^1^H, ^13^C HMBC NMR spectrum of Scotch Whisky


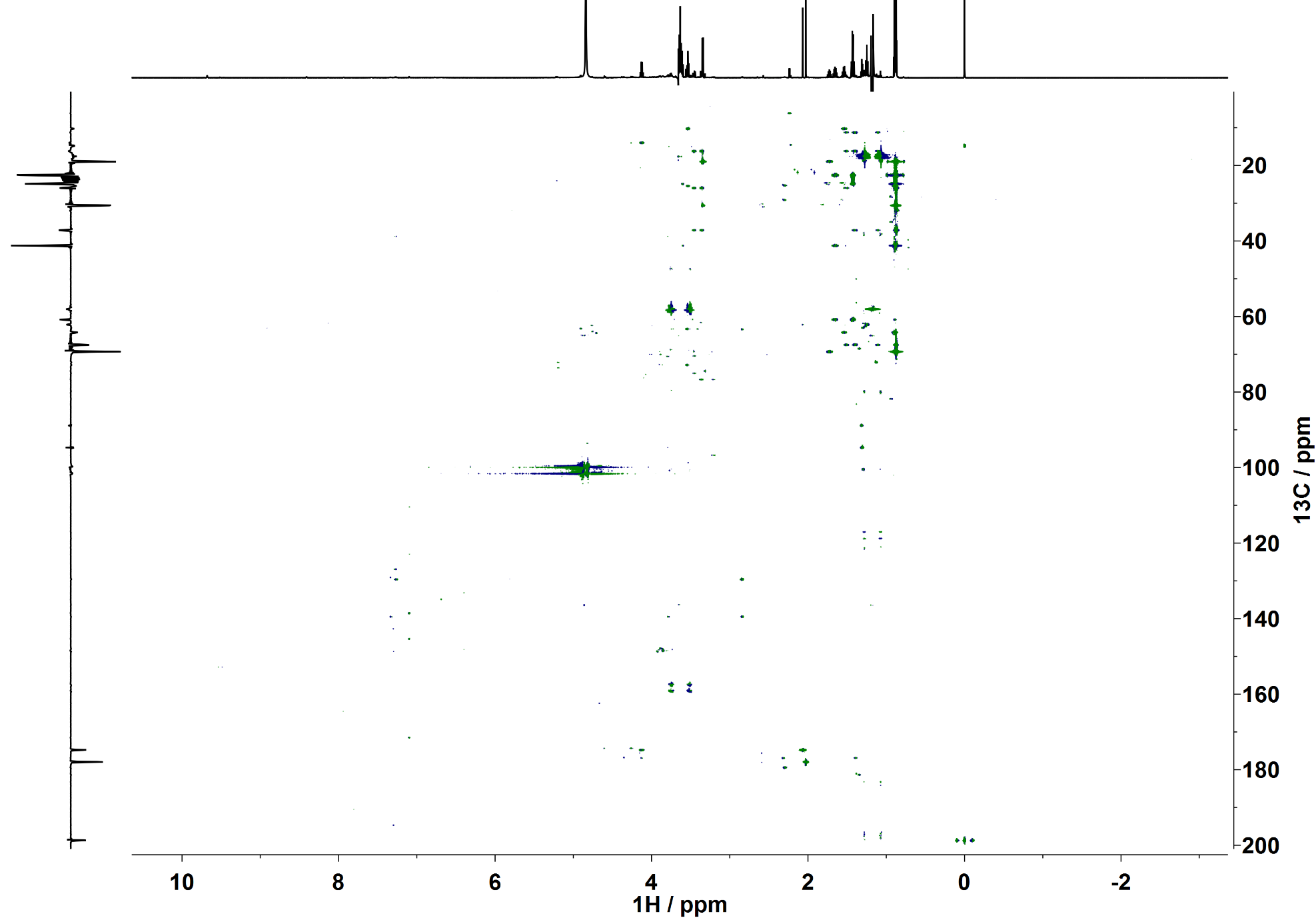


Figure 15 - 2D ^1^H, ^13^C HMBC NMR spectrum of Scotch Whisky with t_1_ noise digitally removed using MestreNova 11
